# Chemical Fingerprinting for Authentication and Compositional Characterization of *Cocos nucifera* Bee Pollen

**DOI:** 10.1021/acsomega.6c07697

**Published:** 2026-10-05

**Authors:** Girliane R. Silva, Déborah M. N. Sousa-Fontoura, Rodolfo F. Alves, Francisco A. R. Santos, Celso A. Camara, Lucas H. N. Sousa, Elenilson G. A. Filho, Eva M. S. Silva, Tania M. S. Silva

**Affiliations:** † Laboratório de Bioprospecção Fitoquímica, Departamento de Química, 67744 Universidade Federal Rural de Pernambuco, 52171-900, Recife, PE, Brazil; ‡ Laboratório de Micromorfologia Vegetal, Departamento de Ciências Biológicas, Universidade Estadual de Feira de Santana, 44036-900, Feira de Santana, BA, Brazil; § Instituto de Química, 28123 Universidade Federal do Rio Grande do Norte, 59078-970, Natal, RN, Brazil; ∥ Departamento de Engenharia de Alimentos, 28121 Universidade Federal do Ceará, Campus do Pici, CEP 60440-900, Fortaleza, CE, Brazil; ⊥ Laboratório de Entomologia, Apicultura e Meliponicultura, Universidade Federal do Vale de São Francisco, 56300-990 Petrolina, PE, Brasil

## Abstract

Bee pollen produced by *Apis mellifera* is formed by the agglutination of floral pollen grains with nectar and bee secretions. Monofloral or dominant bee pollen generally exhibits a more consistent chemical composition and quality. In this study, coconut (*Cocos nucifera*) bee pollen samples collected monthly for 13 months in Maraú, Bahia, Brazil were comprehensively characterized. Palynological analysis confirmed the predominance of coconut pollen throughout the sampling period. Physicochemical parameters met Brazilian quality standards for bee pollen. Chemical profiling included the determination of sugars, free amino acids, and minerals, revealing glucose and fructose as the main sugars and potassium, calcium, and magnesium as the predominant minerals. The major flavonoid, identified as a characteristic marker, was isolated and structurally characterized as isorhamnetin-3-*O*-neohesperidoside, also detected in *C*. *nucifera* floral pollen. Multivariate (PCA) and univariate (ANOVA) analyses indicated limited compositional variability, mainly associated with seasonal and environmental factors influencing the pollen collection.

## Graphical abstract

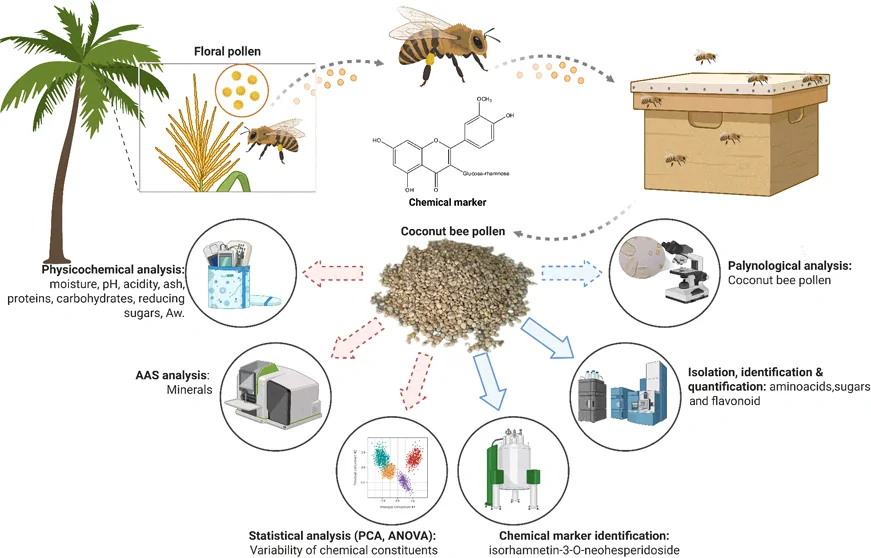

## 1 Introduction

Bee-derived products, such as bee pollen, propolis, honey, royal jelly, and bee bread, have been used by humans since antiquity because of their recognized biological and health-promoting properties.^1^ Among these products, bee pollen has emerged as an important nutritional and functional food source. It is widely employed as an apitherapeutic agent and dietary supplement owing to its high protein content and favorable amino acid composition.^2–4^ In recent years, its consumption has increased among athletes, who use bee pollen as a highly nutritious food to support and potentially enhance their physical performance.^5^

Bee pollen is a complex natural matrix that contains abundant macronutrients, including carbohydrates (4–85%), proteins (2–60%), lipids (1–20%), and fibers (0.3–20%).^6–8^ In addition, it contains minor constituents such as minerals, including calcium, manganese, magnesium, phosphorus, selenium, zinc, copper, iron, potassium, and sodium,^9,10^ trace elements, vitamins (β-carotene, thiamine, riboflavin, niacin, pantothenic acid, pyridoxine, ascorbic acid, biotin, folic acid, and tocopherol),^11^ carotenoids, phenolic compounds, flavonoids,^12^ sterols, and terpenes.^13^ However, the qualitative and quantitative composition of bee pollen may vary considerably depending on several factors, including botanical origin, climatic conditions, soil characteristics, beekeeping practices, and storage conditions.^1^ These compositional variations directly influence the nutritional quality and biological properties of food. Several studies have highlighted a wide range of bioactivities associated with bee pollen, including antioxidant,^14^ antidiabetic and antihyperglycemic,^15^ hepatoprotective and nephroprotective,^16^ antiviral,^17^ antimicrobial, antifungal, and anticancer^7,18^ effects, as well as anti-inflammatory,^3,19^ hypolipidemic,^20,21^ and antiallergic^22,23^ properties. Additionally, bee pollen has been reported to exert an anabolic effect on bone metabolism, regulate ovarian function,^24^ and act as an epigenetic modulator.^25^

Honeybees (*Apis mellifera*) and other bee species rely on pollen as their primary source of protein and lipids.^26,27^ These nutrients are essential for larval nutrition and play a fundamental role in the growth and development of bee colonies.^28^ Bees collect pollen grains from the anthers of flowering plants and subsequently mix them with nectar, honey, and salivary gland secretions, forming small granular pellets, known as bee pollens. The color and chemical composition of these pollen pellets depend strongly on their botanical origin, allowing bee pollen to be classified as monofloral or dominant when derived predominantly from a single plant species, or polyfloral when originating from multiple botanical sources.^29^ For commercial harvesting, beekeepers typically use pollen traps installed at the entrances of the beehives.^30^

Owing to its nutritional value and growing commercial demand, a wide variety of bee pollen-based products are currently available in the market, including oral liquids, nutritional bars, tonics, pellets, tablets, capsules, powders, and granules.^31^ Consequently, the establishment of reliable analytical approaches for determining botanical origin and ensuring product authenticity has become increasingly important for quality control and consumer protection.^32^ In this context, the identification of chemical markers associated with specific botanical sources has emerged as a promising strategy to complement traditional palynological analyses.^33^ Although palynological analysis is widely used to determine the botanical origin of bee pollen, the identification of specific chemical markers that can unequivocally confirm the plant source remains limited for several species. In particular, studies integrating chemical profiling with the direct analysis of pollen grains from plant inflorescence are still scarce. Such approaches are essential for establishing robust correlations between plant-derived metabolites and the chemical composition of bee pollen, thereby improving the reliability of the botanical authentication.

Brazil has established official quality standards for bee pollen through the Ministry of Agriculture, Livestock, and Supply (MAPA).^34^ However, further studies are required to better characterize bee pollen produced in different Brazilian regions, particularly with regard to its chemical composition, botanical origin, and potential bioactive compounds. These investigations are essential for improving product standardization, strengthening quality control, and supporting the expanding market for bee-derived products.

This study continues our ongoing investigations on bee products.^4,9,35–42^ In this study, coconut (*Cocos nucifera)* bee pollen samples collected from the municipality of Maraú, Bahia, Brazil, were investigated over a 13-month period through consecutive monthly sampling to evaluate seasonal behavior and compositional variation. To the best of our knowledge, this is one of the first studies to perform a comprehensive year-round characterization of predominantly *Cocos nucifera* bee pollen produced in a tropical Atlantic Forest ecosystem. The samples were characterized through palynological, physicochemical, and chemical analyses, including the determination of sugars, free amino acids, and mineral composition, as well as the isolation and structural identification of major flavonoids. Principal component analysis (PCA) was used to evaluate the variability among samples based on physicochemical parameters, mineral composition, and flavonoid and amino acid profiles. By integrating palynological analysis with detailed chemical characterization and a comparison with flavonoids present in pollen grains directly collected from *Cocos nucifera* inflorescences, this study provides new insights into the identification of potential chemical markers associated with coconut bee pollen. This approach contributes not only to the chemotaxonomic understanding of phenolic compounds in Arecaceae pollen but also to the development of reliable strategies for food authenticity, traceability, and quality control of bee pollen with a predominant botanical pollen profile.

## 2 Materials and Methods

### 2.1 Sample Collection

Bee pollen from *Apis mellifera* was collected in apiaries located in Maraú, Bahia, Brazil (14° S, 39° W; 10 m a.s.l.) (Figure 1). Samples (1.0 kg) were collected once a month between March 2012 and March 2013 from several hives in the same apiary. The collected material was pooled to obtain a representative sample for each month (Table 1). For bee pollen collection, a pollen trap equipped with a retention grid was installed at the entrance of each of the hives. As the bees passed through the device, some of the pollen loads adhered to the corbiculae of the hind legs were retained in the collecting chamber. The fresh bee pollen was dehydrated on trays under air circulation at room temperature, vacuum-packed, and stored under refrigeration, protected from light and moisture, until analysis (Figure 2). The 13-month sampling period was designed to evaluate seasonal variations in the botanical pollen profile and to ensure the representativeness and reliability of the chemical composition data obtained during the study period. The study site is a peninsula surrounded by marine inlets, with vegetation comprising restinga, Atlantic forests, mangroves, and extensive coconut plantations (*Cocos nucifera*).

**Figure 1:**
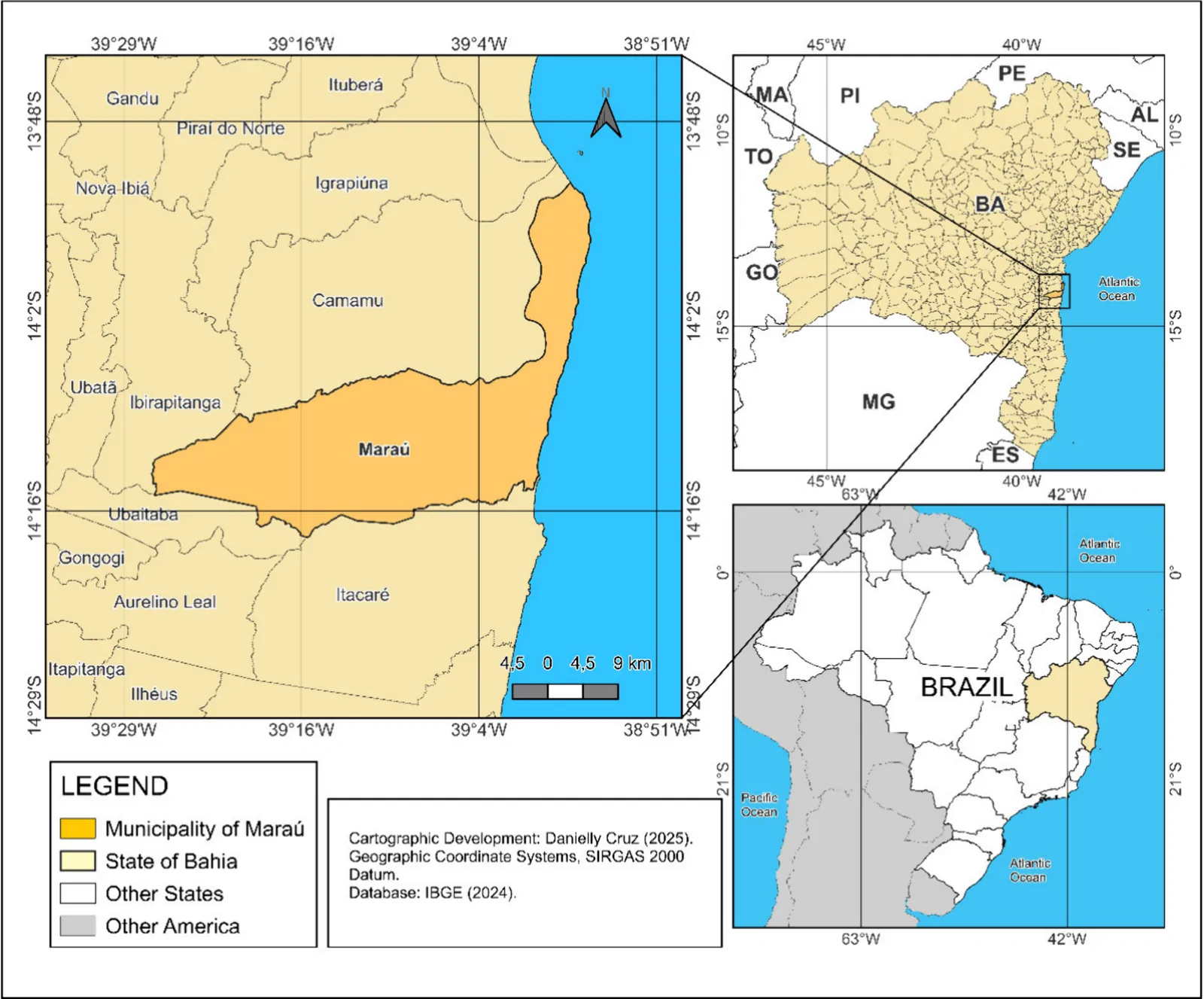
Bee pollen collection region, municipality of Maraú, Northeastern Brazil.

**Table 1:** Sampling Period of 13 Bee Pollen Samples Collected Monthly between March 2012 and March 2013

**Sample**	**Collection period**	**Frequency of** ***Cocos nucifera*** **(%)**	**Frequency of other plant species (%)/Species (Family)**
BP01	March 2012	47.6	22.2/Myrcia (Mirtaceae)
BP02	April 2012	45.2	32.4/Alagoptera (Arecaceae)
BP03	May 2012	63.3	32.1/Alagoptera (Arecaceae)
BP04	June 2012	56.7	21.7/Myrcia (Mirtaceae)
BP05	July 2012	28.4	40.9/Alagoptera (Arecaceae)
BP06	August 2012	43.1	31.2/Mikania (Asteraceae)
BP07	September 2012	64.8	28.2/Mikania (Asteraceae)
BP08	October 2012	41.0	43.5/Mikania (Asteraceae)
BP09	November 2012	50.5	17.6/Clidemia (Melastomataceae)
BP10	December 2012	41.8	25.7/Myrcia (Mirtaceae)
BP11	January 2013	50.8	38.7/Myrcia (Mirtaceae)
BP12	February 2013	53.4	38.6 Byrsonima (Malpighiaceae)
BP13	March 2013	39.7	23.8 Myrcia (Mirtaceae)

**Figure 2:**
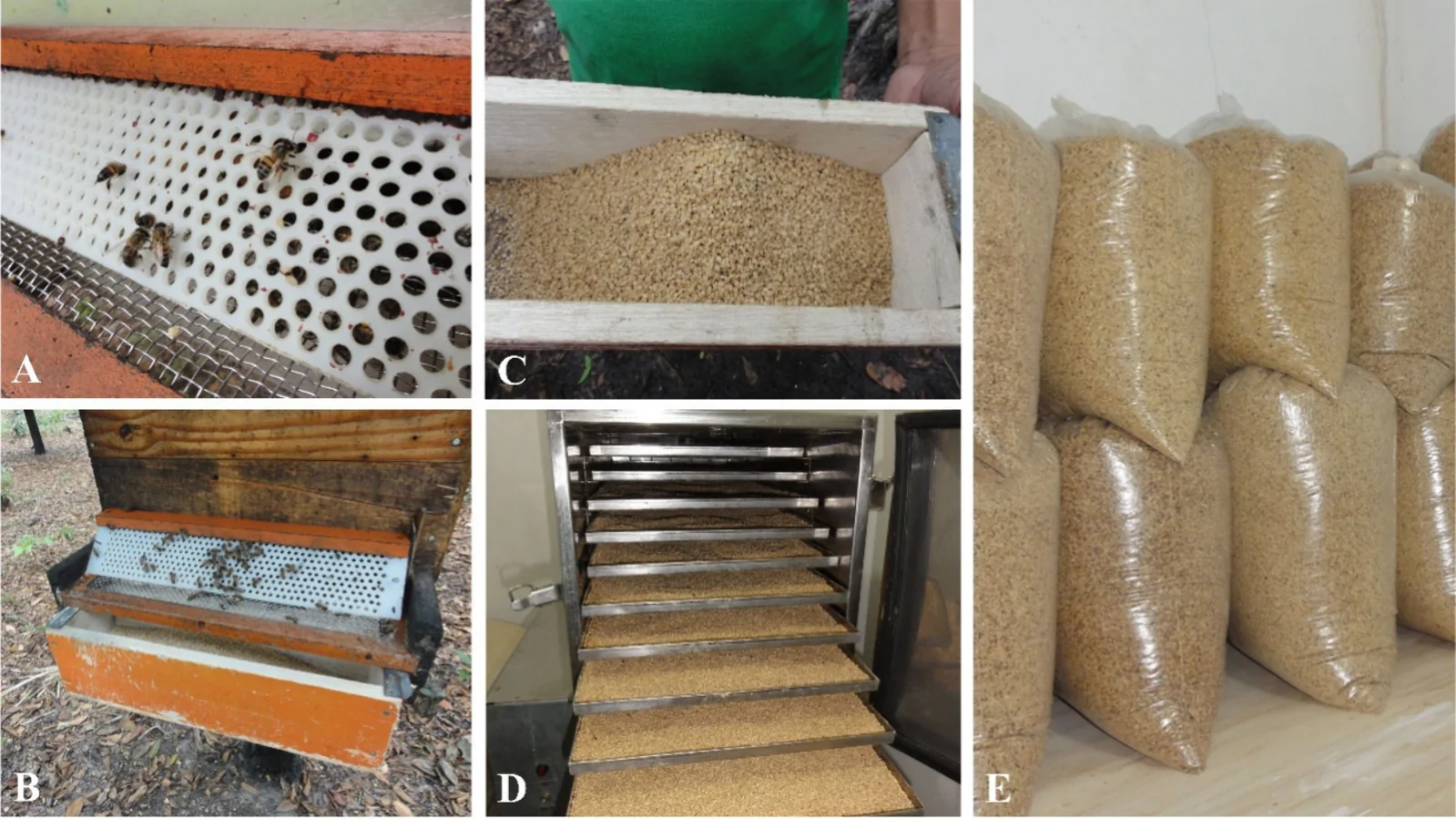
Collection and processing of coconut bee pollen: A and B. Pollen trap; C. Fresh bee pollen; D. Bee pollen drying; E. Storage of dehydrated bee pollen.

### 2.2 Palynological Analysis

Palynological analyses were performed according to ref^43^ with modifications proposed by Novais et al.^44^ Pollen sediments were subjected to acetolysis^45^ and identified by comparison with reference slides from the laboratory collection where voucher specimens had been deposited. Pollen types were classified according to their frequency as very frequent (>50%), frequent (20–50%), infrequent (10–20%), or rare (<10%).^46^

### 2.3 Physicochemical Analysis

Physicochemical analyses were performed using an analytical balance (AW220, Shimadzu, Kyoto, Japan), infrared moisture balance (ID50, Marte, Pernambuco, Brazil), pH meter (TEC-3MP, Tecnal, São Paulo, Brazil), muffle furnace (1P 7000, Edgcon, São Paulo, Brazil), digestion block (TE-040/25GE, Tecnal, São Paulo, Brazil), micro-Kjeldahl apparatus (TE-0363, Tecnal, São Paulo, Brazil), and a water activity analyzer (AquaLab 4TE, Decagon Devices, São Paulo, Brazil).

The bee pollen samples were subjected to the following physicochemical analyses: moisture (%), pH, and free acidity (mEq).kg^1–^), Ash Content (%), Lipid Content (%), Total Protein Content and Carbohydrate Content (%), Reducing Sugar Content (%), and Water Activity (Aw).^33,47^ The analytical methodologies followed the physical-chemical methods for food analysis of the Adolfo Lutz Institute.^48^

### 2.4 Mineral Content

Mineral determination was performed using a sequential flame atomic absorption spectrometer (AA240FS-Fast Sequential, Varian, Mulgrave, Australia) equipped with a deuterium lamp for background correction. Hollow cathode lamps were used as radiation sources for each element, and an air-acetylene flame was employed for all measurements. Hydride generation was performed using a vapor-generation accessory (VGA-77, Varian, Mulgrave, Australia). The samples were digested with nitric acid using a microwave digestion system (Mars Xpress, CEM, São Paulo, Brazil).

The pollen samples (1.0 g) were digested and subsequently diluted (1:1) with distilled water before the analysis. Calibration curves with five concentrations for the metals (Ca, Cu, Fe, K, Mg, Mn, Na, Zn, and Se) were constructed. For the analysis of selenium, which was present at lower concentrations, atomic absorption spectrometry with hydride generation was used.^9^

### 2.5 Amino Acid and Sugar Analysis

Bee pollen samples (2.0 g) were extracted by using distilled water in an ultrasonic bath for 30 min. The aqueous extracts were subsequently lyophilized and used to determine free amino acids and sugars.

Free amino acids were analyzed by high-performance liquid chromatography with diode array detection (HPLC-DAD) after derivatization with the AccQ•Tag reagent, following the manufacturer’s protocol (Waters Corporation, Milford, MA, USA). The analysis was performed using an AccQ•Tag amino acid analysis column (3.9 × 150 mm, 4 μm, 60 Å), operated at 37 °C with a flow rate of 1.0 mL min^–1^. The mobile phases consisted of AccQ•Tag Eluent A, acetonitrile (Eluent B), and HPLC-grade water (Eluent C). Detection was performed using a UV detector at 254 nm.^49^ Seventeen amino acids were quantified: aspartic acid (Asp), serine (Ser), glutamic acid (Glu), glycine (Gly), histidine (His), arginine (Arg), threonine (Thr), alanine (Ala), proline (Pro), cysteine (Cys), tyrosine (Tyr), valine (Val), methionine (Met), lysine (Lys), isoleucine (Ile), leucine (Leu), and phenylalanine (Phe).

Glucose and fructose were determined using high-performance liquid chromatography coupled with a refractive index detector (HPLC-RID). Separation was performed using a Rezex RCM Monosaccharide Ca^2+^ (8%) column (300 mm × 7.8 mm, 8 μm; Phenomenex). The column temperature was maintained at 85 °C, with a flow rate of 0.6 mL min^–1^. The mobile phase consisted of water containing 5% methanol and was run in an isocratic mode.^49^

### 2.6 Isolation of the Major Flavonoid from Cocos nucifera Bee Pollen and Extraction of Cocos nucifera Floral Pollen

A powdered pollen sample (300.0 g) was extracted using ethanol/water (8:2) in an ultrasonic bath. The extractive solution was concentrated under reduced pressure using a rotary evaporator to yield 150.0 g of a crude ethanolic extract. An aliquot of this extract (5.0 g) was dissolved in 8.0 mL of methanol–water (7:1, v/v; pH 2) and subjected to solid-phase extraction (SPE-C18) using methanol and ethyl acetate as eluents. The methanolic fraction (1.7 g) was purified further using preparative HPLC-DAD. This procedure was repeated for all 13 pollen samples, and the methanolic fraction was subjected to HPLC-DAD-qTOF-MS^E^ analysis. Chromatographic separation of the flavonoid was performed using a preparative column (Hydro-RP Synergy 250 mm × 21.2 mm, 4 μm, Phenomenex) maintained at 35 °C. The mobile phase consisted of ultrapure water (eluent A) and methanol (eluent B), delivered at a flow rate of 15 mL min^–1^. The gradient program was as follows: 0–10 min, 40–55% B; 10–15 min, 55–100% B; 15–18 min, 100% B. Chromatograms were monitored at 320 nm. A total of 177.6 mg of the major flavonoid was isolated from the extract. The compound was identified using ^1^H and ^13^C nuclear magnetic resonance (NMR) spectroscopy and high-resolution mass spectrometry (HRMS). Nuclear magnetic resonance (NMR) spectra were recorded on a Bruker DPX-300 spectrometer operating at 300 MHz for ^1^H and 75 MHz for ^13^C. The sample was dissolved in DMSO-*d*_6_, and the chemical shifts (δ) were expressed in parts per million (ppm) relative to tetramethylsilane (TMS) as the internal reference. The coupling constants (J) are reported in Hertz (Hz).

The extraction of *C. nucifera* floral pollen followed the same extraction procedure described above. Floral pollen (531.2 mg) was separated from the flowers and ground. After solvent evaporation, 24.2 mg of the hydroethanolic extract was obtained.

### 2.7 Quantification of the Major Flavonoid in Apis mellifera Bee Pollen and in Pollen Grains Collected from Cocos nucifera Inflorescences

The major flavonoid isolated in this study was quantified in 13 bee pollen samples as well as in *Cocos nucifera* floral pollen. Hydroethanolic extracts were dissolved in methanol and acidified water (1:1, v/v) to a final concentration of 5 mg mL^–1^ and filtered prior to analysis. Chromatographic separation was performed using a column (Synergy Hydro-RP 250 mm × 4.6 mm, 5 μm; Phenomenex). The column temperature was maintained at 40 °C, with a flow rate of 1.0 mL min^–1^. The mobile phase consisted of water (eluent A) and methanol (eluent B), both of which were acidified with 1% formic acid. The gradient program was as follows: 0–10 min, 40–55% B; 10–15 min, 55–100% B; 15–18 min, 100% B; and 18–25 min, 40% B. Quantification of the major flavonoid was performed using an external calibration curve constructed with standard solutions at concentrations of 80, 60, 40, 20, 10, and 5 μg mL^–1^. The injection volumes for both the standards and samples were 5 μL. The chromatograms were monitored at 320 nm. The limit of detection (LOD) was determined at a signal-to-noise (S/N) ratio of 3.3, and the limit of quantification (LOQ) was determined at an S/N ratio of 10.

### 2.8 Statistical Analysis

Data are presented as the mean ± standard deviation of three replicates for each sample. Statistical analyses were performed using GraphPad Prism 5.0 (DEMO) and one-way ANOVA followed by Tukey’s test at a significance level of *p* < 0.05.

#### 2.8.1 Multivariate Statistical Analysis

Unsupervised multivariate statistical analyses by principal component analysis (PCA) were performed to understand the variability of the following data in different pollen samples: physicochemical parameters (protein, lipids, ashes, carbohydrates, reducing sugar, moisture, water activity, pH, free acidity, and percentages of glucose and fructose); concentrations of minerals (calcium, copper, iron, potassium, magnesium, manganese, sodium, zinc, and selenium); and concentrations of amino acids (aspartate, serine, glutamic acid, glucose, histidine, arginine, threonine, alanine, proline, tyrosine, valine, methionine, lysine, isoleucine, leucine, and phenylalanine).

A numerical matrix was created for each type of data set (physicochemical, minerals, and amino acids), which were separately imported by the PLS-Toolbox software (version 8.6.2, eigenvector Research Incorporated, Manson, WA, USA, 2018) and autoscaled. For PCA evaluation, the singular value decomposition (SVD) algorithm was applied to decompose these complex matrices into scores, loadings, and modeling residues. The most relevant information regarding pollen variabilities was achieved using the first two principal components (2 PC), using the cross-validation methods by Venetian Blinds under 95% confidence.^50,51^

#### 2.8.2 Univariate Statistical Analysis

The combined uncertainties of the amounts of physicochemical parameters, concentrations of minerals, concentrations of amino acids, and concentrations of flavonoids in pollen with the percentage of *C. nucifera* pollen were estimated on the basis of the sampling replicates. Then, the respective data sets were subjected to single-factor analysis of variance (ANOVA) using the Origin 9.4 software, under the following parameters to statistically certify the variability between the pollens: significance level of 0.05; comparison of means using the Tukey test; and Levene to test the homogeneity of variance.

## 3 Results and Discussion

### 3.1 Botanical Origin of Bee Pollen

Table 1 shows the results of the palynological analyses for the 13 bee pollen samples analyzed, indicating the frequency of coconut pollen grains (*Cocos nucifera*, Arecaceae), which ranged from 28.4 to 64.8%. Figure 3 shows a micrograph of *C. nucifera* pollen from one of the analyzed samples. The apiary from which the samples were collected is located near the sea in a large coconut plantation area, where flowering occurs throughout the year, with a more intense blooming period between January and April.^52^ Coconut palms are an important source of pollen and proteins for *A. mellifera* bees in that region.

**Figure 3:**
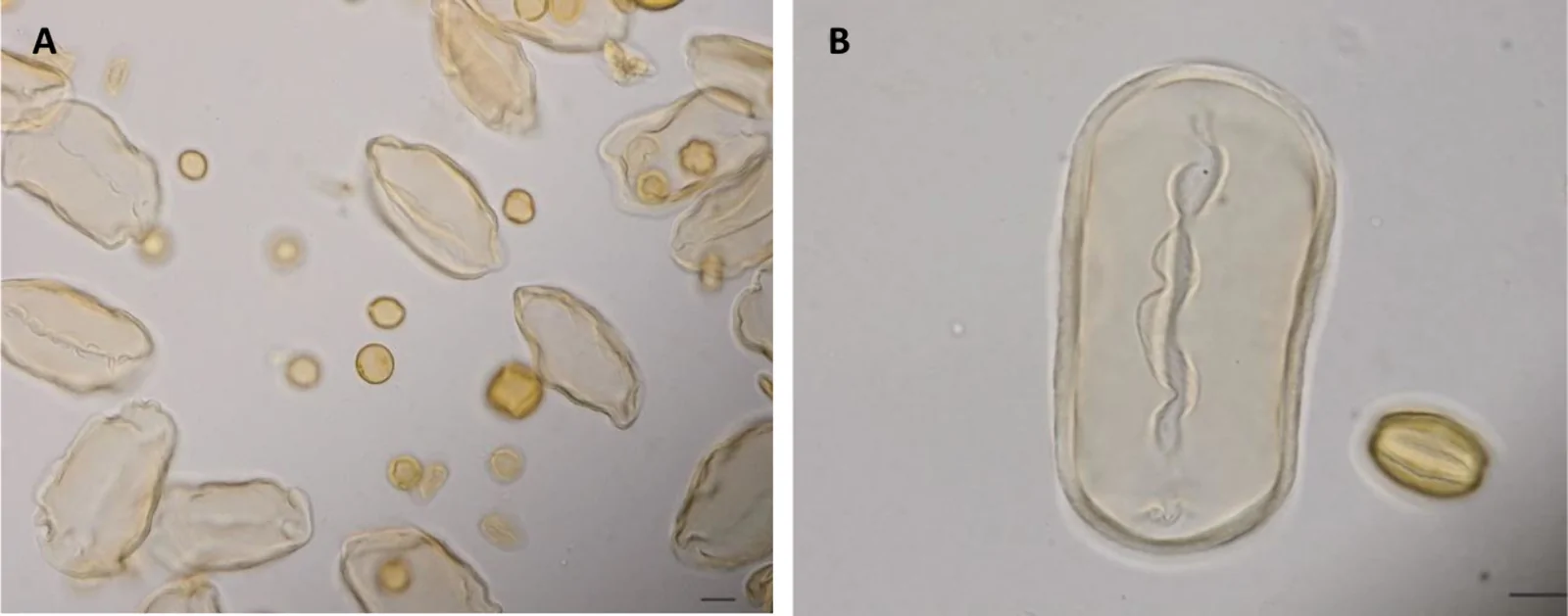
Light micrographs of the bee pollen. (A) Mixed pollen grains with a predominance of larger grains. (B) *Cocos nucifera* pollen grain.

All analyzed bee pollen samples contained coconut pollen grains (*C. nucifera*), which were classified as frequent (BP05, BP06, BP08, BP10, and BP13) or very frequent (BP01, BP02, BP03, BP04, BP07, BP09, BP11, and BP12). Samples containing more than 45% coconut pollen grains were classified as monofloral or dominant. Although other plant species occur in the surrounding area, bees predominantly collect pollen from coconut palms because of their greater availability and continuous flowering throughout the year.^53^

Monofloral bee pollen is particularly relevant for product standardization as it tends to have a more consistent chemical composition and physicochemical characteristics. Moreover, the botanical and geographical origins are key parameters for assessing the quality and authenticity of bee pollen as a food product.^54,55^ Similar results were reported for bee pollen collected near coconut plantations in the municipality of Canavieiras (Bahia, Brazil), where monofloral coconut pollen with up to 99% *C. nucifera* pollen grains was observed.

### 3.2 Physicochemical Characterization of Bee Pollen

The analyzed bee pollen samples consisted of heterogeneous grains with variable shapes and sizes, generally tending toward a spherical morphology. The samples exhibited a light-yellow color, characteristic aroma, and sweet taste. No impurities, such as bee bodies, wax residues, plant fragments, or other foreign materials, were observed, indicating appropriate harvesting and sample handling.

The physicochemical composition of *Apis mellifera* bee pollen is shown in Table 2. On a dry basis, the samples contained 22.23 ± 0.88% protein, 2.59 ± 0.46% lipids, 3.69 ± 0.30% ash, and 71.41 ± 1.11% carbohydrates. Additionally, the samples contained 38.37 ± 4.36% reducing sugars, 6.87 ± 0.47% moisture, water activity of 0.34 ± 0.03, pH of 4.47 ± 0.25, and free acidity of 477.97 ± 77.28 mequiv.kg^–1^. All physicochemical parameters were within the limits established by Brazilian legislation for bee pollen quality.^34^

**Table 2:**
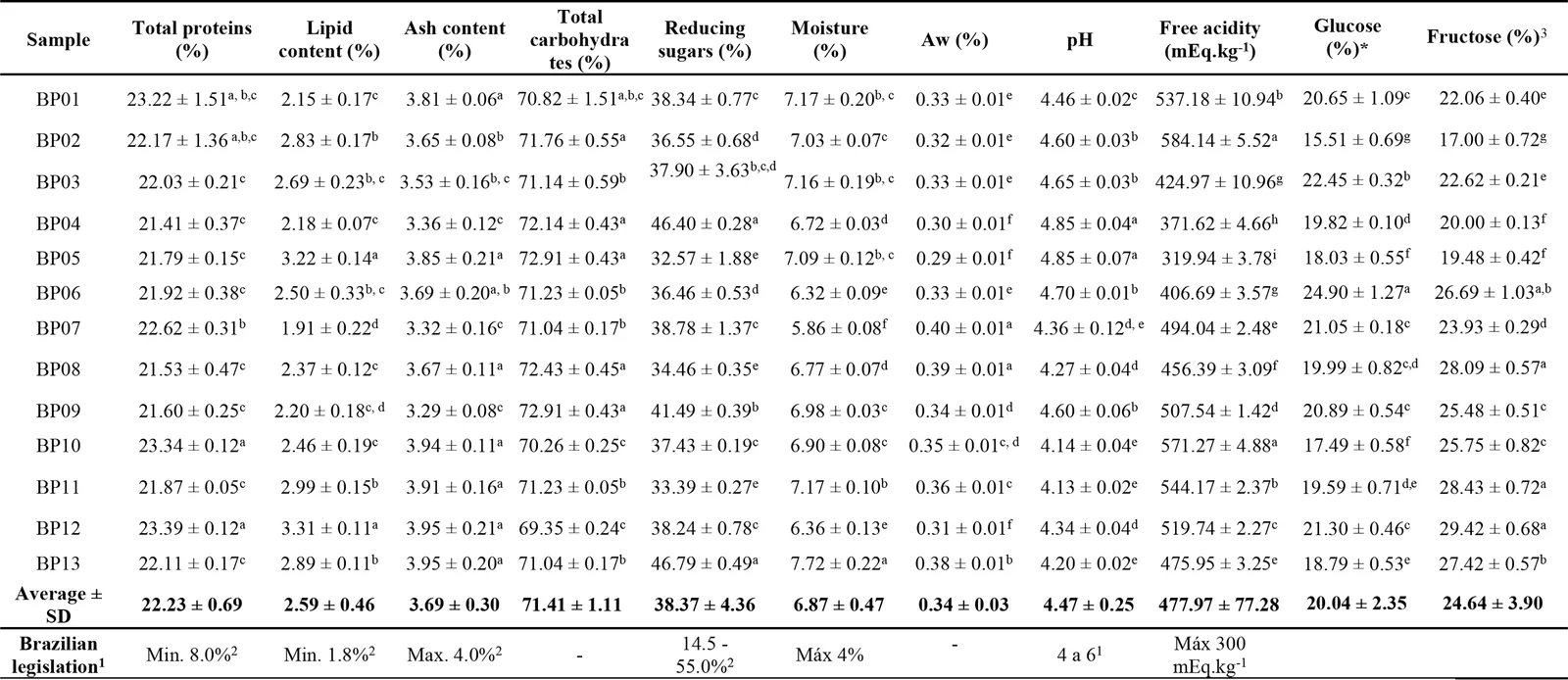
Physicochemical Parameters of Coconut (*Cocos nucifera*) Bee Pollen Produced by *Apis mellifera* and Collected Monthly in 2012–2013 in Maraú, Bahia, Brazil

* Analyzed by HPLC-RID.

1 Limits according to Technical Regulation on the Identity and Quality of Bee Pollen (Normative Instruction n° 3, 2001) of the Ministry of Agriculture, Livestock and Supply - MAPA.

2 m/m, on a dry basis.

a,b,c The same letters in the same column indicate that the results did not show a significant difference between the months (*p* > 0.05).

3 *n* = 2.

The protein content observed in the analyzed samples was consistent with previously reported values for bee pollen of different botanical origins. According to Komosinska-Vassev et al.,^56^ bee pollen contains an average of 22.7% protein, including approximately 10.4% essential amino acids, confirming its recognized nutritional value. Similar protein ranges (20–25%) have been reported for bee pollen from several geographic regions, suggesting that the samples analyzed in this study present a typical protein profile.

The lipid content (2.59%) was comparable to that reported in the literature for bee pollen collected in areas dominated by *C. nucifera*. In a study conducted by Negri et al.^57^ in coconut-producing regions of Bahia (Brazil), bee pollen samples presented a lipid content of 2.7%, which is very similar to that observed in the present work. Lipids in bee pollen mainly consist of fatty acids and phospholipids, which contribute to its nutritional and functional properties.^58^

The ash content, which reflects the mineral fraction of bee pollen, was slightly higher than the 2.54% reported for coconut-dominant bee pollen collected in the municipality of Canavieiras (Bahia, Brazil).^56^ Variations in mineral content may be influenced by several factors, including the mineral accumulation capacity of the plant species, geographical origin, soil characteristics, and floral diversity.^59,60^

Carbohydrates were the major fraction of the analyzed bee pollen samples, accounting for more than 70% of dry matter. A significant portion of these carbohydrates originates from nectar or honey incorporated by bees during the formation of pollen pellets.^61^ Consequently, the carbohydrate profile of bee pollen may reflect not only the botanical origin of the pollen grains but also the biochemical processing performed by the bees.

Moisture content and water activity are critical parameters that affect the stability, organoleptic properties, and shelf life of bee pollen.^62^ While the moisture content represents the total amount of water present in a product, the water activity reflects the fraction of free water available for microbial growth.^54^ The relatively low water activity observed in the analyzed samples indicates favorable conditions for product stability and a reduced susceptibility to microbial growth during storage.

Free acidity is another important indicator used to assess the conservation status and overall quality of the food products. Elevated acidity values may be associated with the presence of organic acids naturally occurring in pollen or with biochemical transformations that occur during storage. Therefore, adequate control of processing stages, particularly collection, drying, and storage conditions, is essential to maintaining the quality of bee pollen. Among the available drying methods, freeze-drying has been reported to be one of the most effective techniques for preserving the chemical composition, antioxidant capacity, and biological properties of bee pollen.^63^

To further investigate the variability of physicochemical parameters among the analyzed pollen samples, we performed principal component analysis (PCA). The PCA results are presented in Figure 4, which shows the score plot (a) and the loading plot (b). A clear clustering was observed for samples BP10, BP11, BP12, and BP13, located at positive PC1 scores, associated with higher values of protein, free acidity, ash, fructose, and water activity and lower values of total carbohydrates, reducing sugars, and pH. In contrast, samples BP04 and BP05 were located at the most negative PC1 scores, exhibiting the opposite behaviors for these variables. Samples BP03 and BP02 exhibited intermediate characteristics between those of the two groups.

**Figure 4:**
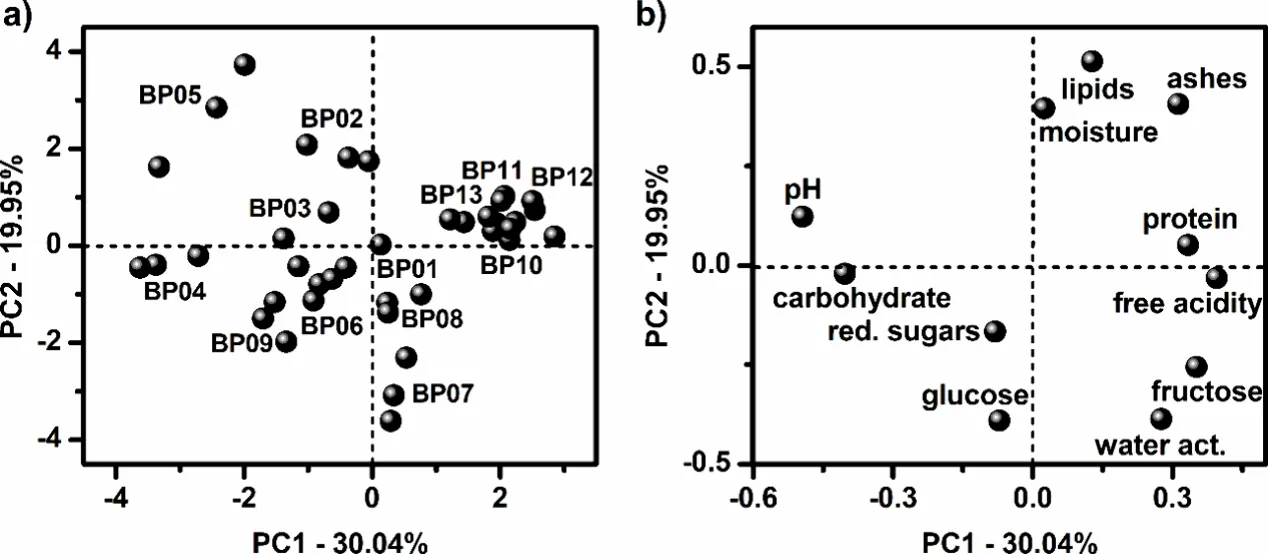
a) PC1 × PC2 scores from the evaluation of the physicochemical parameter variability based on the pollen samples; b) respective PC1 × PC2 loadings.

To complement the multivariate analysis, univariate statistical analysis (ANOVA) was performed to evaluate the differences in physicochemical parameters between the pollen samples. The results are presented in Table 2, where different letters above the bars indicate statistically significant differences between the samples. The ANOVA results corroborated the trends observed in PCA, confirming the variability among samples. For instance, samples BP04 and BP05 presented significantly higher values of total carbohydrates and pH, whereas lower values of protein and free acidity were observed.

Overall, the combination of multivariate (PCA) and univariate (ANOVA) statistical approaches provided a comprehensive evaluation of the physicochemical variability of the bee pollen samples, revealing that differences in composition may be associated with variations in environmental conditions and seasonal factors influencing the pollen collection.

### 3.3 Mineral Composition and Variability of Bee Pollen

The mineral composition of *Apis mellifera* bee pollen is shown in Table 3. Among the analyzed elements, the predominant minerals in the bee pollen samples were potassium (K) (812.61 ± 57.67 mg 100 g^–1^), calcium (Ca) (235.44 ± 22.36 mg 100 g^–1^), and magnesium (Mg) (74.92 ± 4.16 mg 100 g^–1^). The remaining minerals, including Na, Mn, Zn, Fe, Cu, and Se, were detected in comparatively lower concentrations.

**Table 3:**
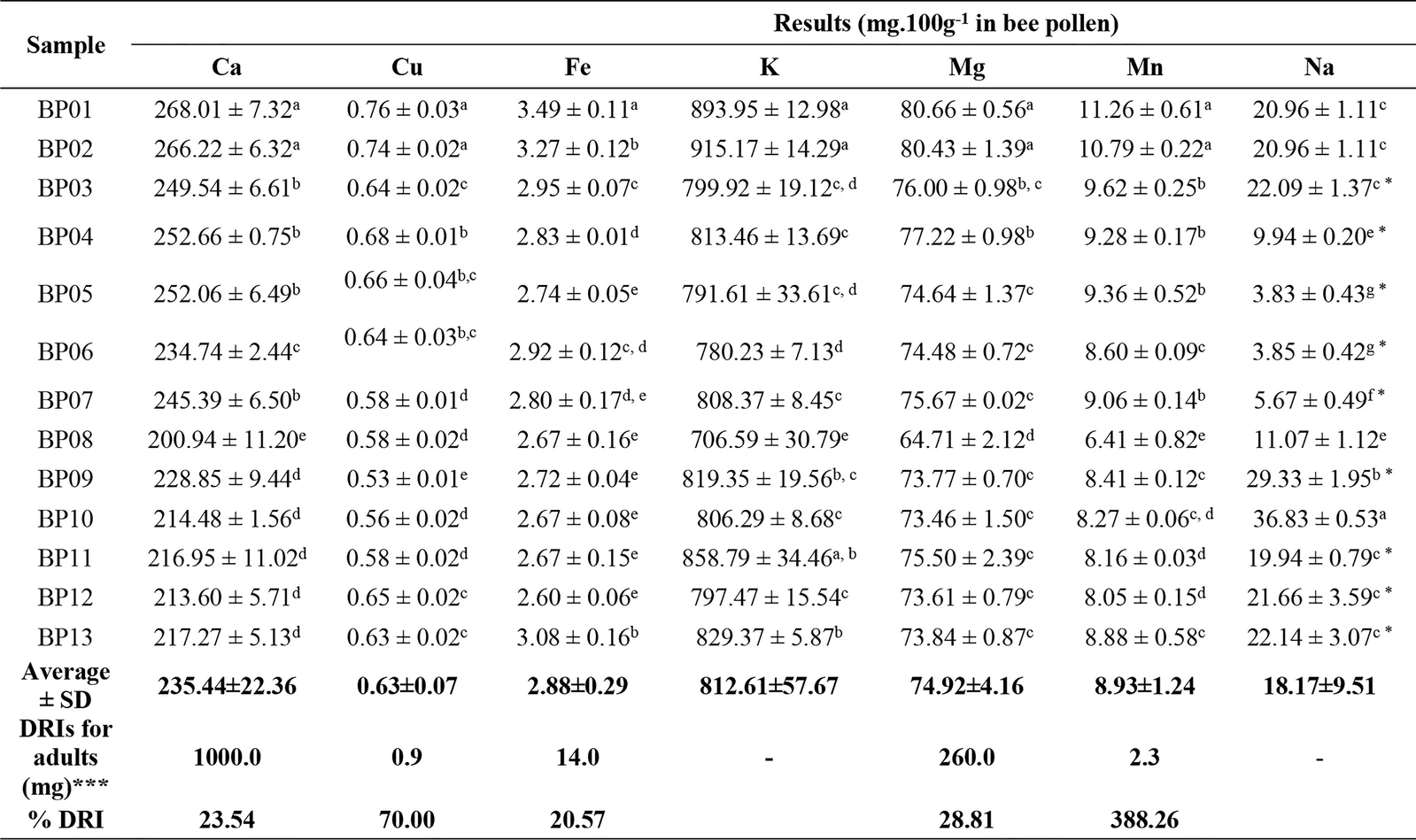
Mineral Composition of Coconut (*Cocos nucifera*) Bee Pollen Produced by *Apis mellifera* (mg/100 g ± SD) and Dietary Reference Intakes (DRIs) for Adults

* *n* = 2.

** Concentração em μg/100g de pollen.

*** Dietary reference intakes for adults (RDC 269/2005).

The predominance of potassium, calcium, and magnesium observed in this study is consistent with previous reports describing the mineral profiles of bee pollen. In general, K, P, Ca, and Mg are the most abundant minerals in bee pollen, as reported in several studies conducted with samples from different geographical regions.^2,3,10,59^ In Brazil, bee pollen samples collected from the Northeast region have been reported to contain relatively high mineral concentrations compared with samples from other regions, which may be related to regional climatic conditions and floral diversity.^10^ In addition, the mineral composition of bee pollen can vary significantly depending on its botanical origin, geographical location, and environmental conditions.^2^

Principal component analysis (PCA) was used to investigate the variability in the mineral composition among the pollen samples. The PCA results are presented in Figure 5, showing the score plot (a) and loading plot (b) for the minerals Ca, Cu, Fe, K, Mg, Mn, Na, Zn, and Se. PCA revealed the formation of three main clusters. The first group, located at positive PC1 scores (samples BP01 and BP02), was associated with higher concentrations of Ca, Cu, Mn, Zn, Fe, Mg, and K. The second group, positioned at intermediate PC1 scores and negative PC2 values (samples BP03, BP04, BP05, BP06, and BP07), presented moderate levels of these minerals, particularly Ca and Cu, in the samples. The third group, located at negative PC1 scores (samples BP08, BP09, BP10, BP11, BP12, and BP13), was characterized by higher concentrations of Na and Se.

**Figure 5:**
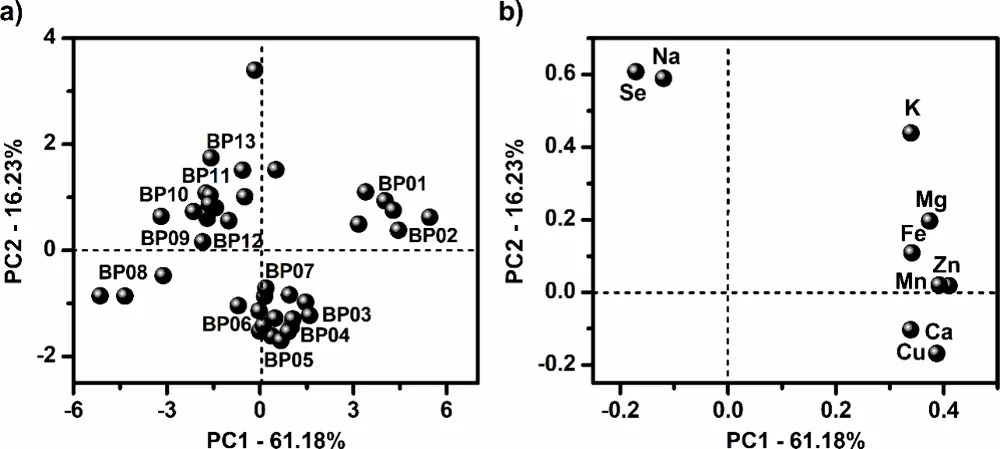
a) PC1 × PC2 scores from the evaluation of mineral variability based on the pollen samples; b) respective PC1 × PC2 loadings.

To further evaluate the differences in mineral composition among the samples, a univariate statistical analysis (ANOVA) was performed (Table 3). The ANOVA results corroborated the PCA findings, confirming higher concentrations of Na and Se in samples BP09, BP11, BP12, and BP13 and elevated sodium levels in samples BP08 and BP09. Interestingly, the lowest sodium concentrations were observed during the period of lower temperatures in the region (May to September 2012), which may be related to the influence of environmental conditions on plant mineral uptake, as sodium absorption by plants tends to increase under higher temperature conditions.^64^ In addition, higher concentrations of Ca, Cu, Fe, K, Mg, Mn, and Zn were detected in samples BP01 and BP02, suggesting that seasonal and environmental factors may influence the mineral accumulation in the pollen collected by bees.

Overall, the combined multivariate (PCA) and univariate (ANOVA) statistical approaches revealed that although the general mineral profile of the analyzed bee pollen samples was consistent with values reported in the literature, noticeable variability existed among samples, likely associated with climatic conditions and seasonal factors influencing pollen production and collection.

### 3.4 Amino Acid Analysis and Variability

The total free amino acid content in the bee pollen samples ranged from 14.67 mg g^–1^ (BP07) to 27.50 mg g^–1^ (BP04) (Table 4). On average, the samples contained 17.49 ± 3.23 mg of free amino acids per gram of pollen, corresponding to 7.86% of the total protein. From a nutritional and functional perspective, the presence of free amino acids is particularly relevant because they are readily available for direct absorption by the human body.

**Table 4:** Amino Acid Composition of Coconut (*Cocos nucifera*) Bee Pollen Produced by *Apis mellifera* ^a^

	**Results (mg·g** ^**–1**^ **of pollen)**													
Amino acids	**BP01**	**BP02**	**BP03**	**BP04**	**BP05**	**BP06**	**BP07**	**BP08**	**BP09**	**BP10**	**BP11**	**BP12**	**BP13**	**Average ± SD**
Asp	0.45 ± 0.01^f^	0.71 ± 0.01^c^	0.44 ± 0.02^f^	0.85 ± 0.02^b^	0.68 ± 0.01^d^	0.39 ± 0.01^g^	0.51 ± 0.03^e^	0.76 ± 0.02^c^	0.78 ± 0.01^c^	0.95 ± 0.03^a^	1.02 ± 0.05^a^	0.86 ± 0.01^b^	0.86 ± 0.02^b^	0.72 ± 0.21
Ser	2.99 ± 0.01^a^	2.07 ± 0.07^c^	2.72 ± 0.09^a^	2.71 ± 0.08^a^	2.22 ± 0.03^b^	1.43 ± 0.01^f^	1.20 ± 0.04^g^	1.37 ± 0.02^f^	1.61 ± 0.01^e^	1.73 ± 0.03^d^	2.06 ± 0.06^c^	2.15 ± 0.03^c^	1.69 ± 0.01^e^	2.05 ± 0.55
Glu	0.40 ± 0.01^g^	0.43 ± 0.02^g^	0.39 ± 0.02^g^	1.62 ± 0.04^a^	0.97 ± 0.03^c^	0.52 ± 0.02^f^	0.67 ± 0.04^e^	0.63 ± 0.04^e^	0.84 ± 0.01^d^	1.00 ± 0.04^c^	1.16 ± 0.03^b^	0.94 ± 0.03^c^	0.84 ± 0.04^d^	0.80 ± 0.35
Gly	0.33 ± 0.01^f^	0.36 ± 0.01^d^	0.32 ± 0.01^f^	0.66 ± 0.02^a^	0.45 ± 0.01^b^	0.38 ± 0.01^c^	0.39 ± 0.03^c^	0.22 ± 0.03^g^	0.35 ± 0.01^e^	0.36 ± 0.01^e^	0.44 ± 0.02^b^	0.36 ± 0.01^e^	0.40 ± 0.01^c^	0.39 ± 0.10
His	2.00 ± 0.08^c^	1.59 ± 0.04^e^	1.97 ± 0.02^c^	2.83 ± 0.04^a^	2.41 ± 0.10^b^	1.71 ± 0.03^d^	1.36 ± 0.01^f^	1.40 ± 0.04^f^	1.36 ± 0.01^f^	1.26 ± 0.03^g^	1.60 ± 0.01^d^	1.61 ± 0.01^d^	1.15 ± 0.02^h^	1.79 ± 0.51
Arg	1.02 ± 0.03^c^	0.89 ± 0.02^d^	0.90 ± 0.02^d^	1.66 ± 0.02^a^	1.39 ± 0.04^b^	0.95 ± 0.05^c, d^	0.40 ± 0.01^g^	0.66 ± 0.03^f^	0.95 ± 0.01^c^	0.80 ± 0.01^e^	0.93 ± 0.02^c^	0.88 ± 0.02^d^	0.81 ± 0.03^e^	0.93 ± 0.32
Thr	0.36 ± 0.02^e^	0.38 ± 0.01^e^	0.32 ± 0.01^f^	0.76 ± 0.04^a^	0.54 ± 0.01^b^	0.41 ± 0.01^c^	0.40 ± 0.01^d^	0.41 ± 0.02^d^	0.44 ± 0.02^c, d^	0.43 ± 0.01^c^	0.51 ± 0.01^b^	0.45 ± 0.01^c^	0.40 ± 0.01^e^	0.46 ± 0.12
Ala	1.30 ± 0.01^e^	1.33 ± 0.02^e^	1.08 ± 0.05^g^	2.39 ± 0.04^a^	1.72 ± 0.03^b^	1.39 ± 0.07^e^	1.18 ± 0.01^f^	1.25 ± 0.06^f^	1.55 ± 0.04^c, d^	1.52 ± 0.03^d^	1.62 ± 0.02^c^	1.50 ± 0.01^d^	1.33 ± 0.02^e^	1.50 ± 0.34
Pro	5.10 ± 0.04^d^	5.20 ± 0.16^d^	5.23 ± 0.06^d^	6.29 ± 0.05^a^	4.76 ± 0.04^e^	4.73 ± 0.16^e, f^	4.59 ± 0.11^f^	6.07 ± 0.10^a, b^	5.20 ± 0.04^d^	5.60 ± 0.06^c^	5.93 ± 0.04^b^	5.58 ± 0.02^c^	5.10 ± 0.01^d^	5.32 ± 0.56
Cys/tyr	0.40 ± 0.01^g^	0.63 ± 0.01^f^	0.77 ± 0.04^c, d^	1.15 ± 0.03^a^	0.97 ± 0.03^b^	0.81 ± 0.01^c^	0.71 ± 0.04^d, e^	0.69 ± 0.03^e^	0.63 ± 0.01^f^	0.66 ± 0.03^e^	0.87 ± 0.06^c^	0.81 ± 0.04^c^	0.65 ± 0.05^e, f^	0.76 ± 0.19
Val	0.53 ± 0.01^g^	0.61 ± 0.01^f^	0.53 ± 0.03^g^	1.11 ± 0.03^a^	0.89 ± 0.01^b^	0.56 ± 0.02^g^	0.52 ± 0.03^g^	0.66 ± 0.01^e^	0.62 ± 0.01^f^	0.72 ± 0.04^d^	0.82 ± 0.02^c^	0.73 ± 0.03^d^	0.64 ± 0.03^e^	0.67 ± 0.17
Met	0.47 ± 0.01^e^	0.35 ± 0.01^h^	0.43 ± 0.02^f^	0.73 ± 0.02^a^	0.64 ± 0.02^b^	0.55 ± 0.01^c^	0.50 ± 0.01^d^	0.38 ± 0.01^g^	0.41 ± 0.01^f^	0.47 ± 0.01^e^	0.46 ± 0.01^e^	0.41 ± 0.03^f, g^	0.42 ± 0.02^f, g^	0.48 ± 0.11
Lys	0.69 ± 0.01^e^	0.75 ± 0.02^d^	0.60 ± 0.01^f^	1.44 ± 0.03^a^	0.98 ± 0.03^b^	0.65 ± 0.02^e^	0.35 ± 0.01^h^	0.47 ± 0.01^g^	0.65 ± 0.01^e^	0.63 ± 0.01^e^	0.88 ± 0.01^b^	0.79 ± 0.01^c^	0.65 ± 0.01^e^	0.76 ± 0.30
Ile	0.27 ± 0.01^g^	0.29 ± 0.02^g^	0.25 ± 0.04^g^	0.74 ± 0.01^a^	0.55 ± 0.02^b^	0.34 ± 0.03^f^	0.38 ± 0.02^e, f^	0.37 ± 0.01^f^	0.40 ± 0.01^e^	0.49 ± 0.01^c^	0.51 ± 0.03^b, c^	0.46 ± 0.03^c^	0.40 ± 0.01^d^	0.43 ± 0.13
Leu	0.73 ± 0.01^h^	0.80 ± 0.01^g^	0.60 ± 0.02^i^	1.55 ± 0.01^a^	1.29 ± 0.02^b^	1.05 ± 0.04^e^	0.96 ± 0.02^f^	0.88 ± 0.02^f^	1.13 ± 0.03^d^	1.13 ± 0.03^d^	1.23 ± 0.01^c^	1.06 ± 0.02^e^	0.95 ± 0.01^f^	1.04 ± 0.24
Phe	0.61 ± 0.02^d^	0.55 ± 0.01^f^	0.51 ± 0.03^f^	0.97 ± 0.04^a^	0.87 ± 0.02^b^	0.72 ± 0.02^c^	0.59 ± 0.02^d, e^	0.62 ± 0.01^d^	0.58 ± 0.01^e^	0.61 ± 0.02^d^	0.69 ± 0.03^c^	0.62 ± 0.02^d^	0.56 ± 0.02^f^	0.67 ± 0.14
Total Free AA	17.65 ± 1.29	16.93 ± 1.28	17.08 ± 1.31	27.50 ± 1.41	21.32 ± 1.10	16.56 ± 1.12	14.67 ± 1.08	16.82 ± 1.42	17.49 ± 1.18	18.33 ± 1.26	20.73 ± 1.24	19.19 ± 1.18	16.76 ± 1.14	17.49 ± 3.23
AA Essentials(%)	32.04	31.39	30.54	36.96	38.15	36.30	34.39	30.74	31.88	31.26	32.20	31.87	30.66	31.88 ± 2.60

a Different letters indicate statistically significant differences between the samples.

Proline was the most abundant free amino acid in the bee pollen samples, with an average concentration of 5.32 ± 0.56 mg g^–1^, representing 30.41% of the total free amino acids content. Among the essential amino acids, histidine was the most abundant (1.79 mg · g^–1^). Other predominant amino acids included serine (2.05 ± 0.55 mg g^–1^) and alanine (1.50 ± 0.34 mg g^–1^), both of which are classified as nonessential amino acids (Table 4). In addition, aspartic acid, glutamic acid, glycine, arginine, threonine, cysteine, tyrosine (coeluted), valine, methionine, lysine, isoleucine, leucine, and phenylalanine were detected in smaller amounts. With the exception of tryptophan, which was not detected in this analysis, all essential amino acids were identified, with concentrations ranging from 0.43 to 1.04 mg g^–1^ pollen. On average, the essential amino acids accounted for 31.88% of the total free amino acid content. These amino acids are required for several physiological processes and must be obtained through diet, as they cannot be synthesized by the human body. Figure 6 shows the chromatographic profile of the standard mixture and representative bee pollen. The predominance of proline (9) was observed, followed by serine (2), which was the second most abundant amino acid in the samples. The regression curves obtained for all amino acid standards showed linear regression coefficients higher than 0.99, indicating the excellent linearity of the analytical method. The limits of detection and quantification were calculated for each amino acid and are listed in Table 5. Proline has also been reported as the predominant amino acid in different bee products in previous studies.^1^ Regarding the essential amino acids, their relative contributions in the analyzed samples ranged from 30.54 to 38.15%, with an average value of 31.88%. These results are consistent with those reported by De-Melo et al.,^65^ who described that essential amino acids in bee pollen samples represent between 12 and 45.02% of the total amino acid content of the samples.

**Figure 6:**
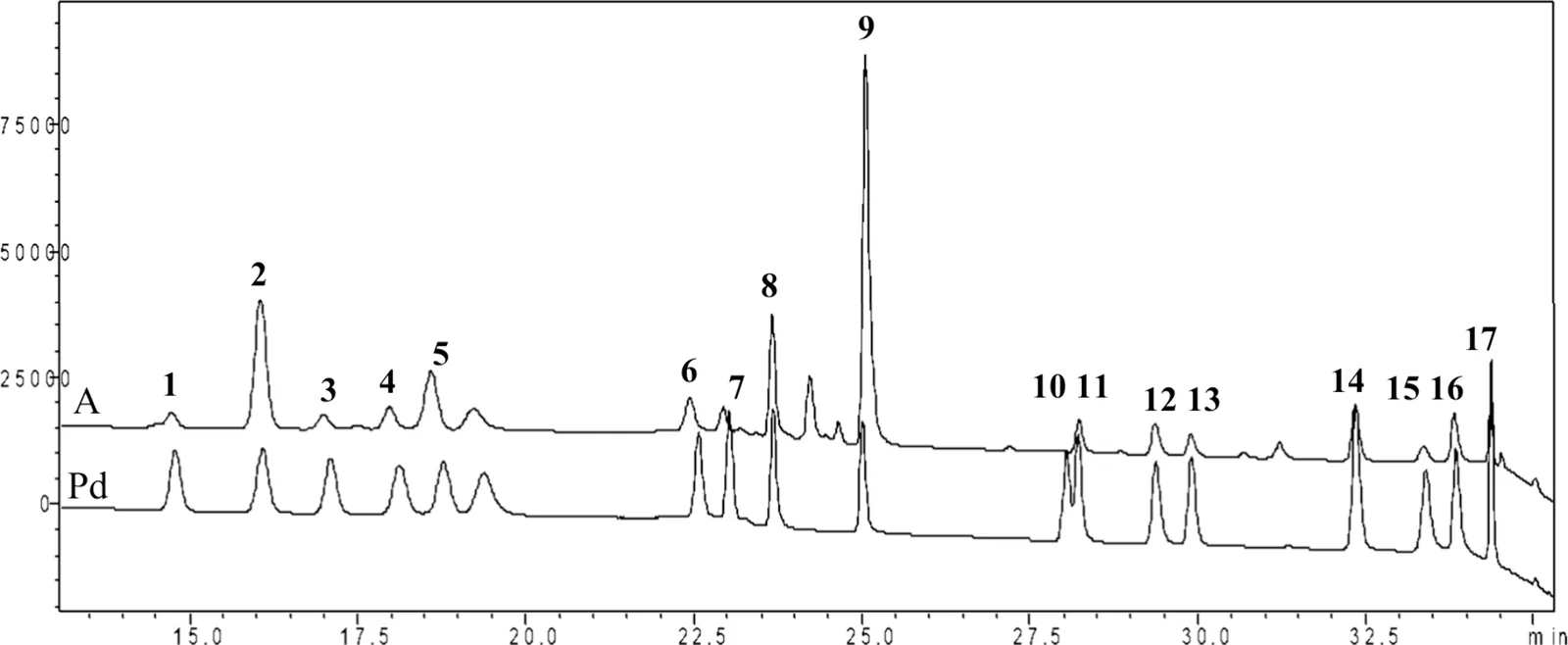
HPLC-DAD chromatograms of the amino acid standard mixture (Pd) and a coconut (*Cocos nucifera*) bee pollen sample produced by *Apis mellifera* (S). Peaks: 1, aspartic acid; 2, serine; 3, glutamic acid; 4, glycine; 5, histidine; 6, arginine; 7, threonine; 8, alanine; 9, proline; 10, cysteine; 11, tyrosine; 12, valine; 13, methionine; 14, lysine; 15, isoleucine; 16, leucine; 17, phenylalanine. Chromatographic conditions: reverse-phase Nova-Pak C18 column (Waters AccQ•Tag, 150 mm × 3.9 mm, 4 μm); column temperature 37 °C; flow rate 1.0 mL min^–1^; injection volume 10 μL. The mobile phase consisted of AccQ•Tag acetate-phosphate buffer (1% eluent A), acetonitrile (eluent B), and ultrapure water (eluent C). The chromatograms were monitored at 254 nm. The gradient program was as follows: 0.01 min, 100% A; 0.5 min, 99% A, 1% B; 18.0 min, 95% A, 5% B; 19.0 min, 91% A, 9% B; 29.5 min, 83% A, 17% B; 33.0 min, 60% B, 40% C; 36.0 min, 100% A; 65.0 min, 60% B, 40% C; 100.0 min, 60% B, 40% C, according to the AccQ•Tag method (Waters manual). HPLC (Shimadzu Prominence LC-20AT, Shimadzu, Kyoto, Japan), two LC-6AT pumps, a DGU-20As degasser, a CTO-20AC oven, and an SPDM-20A diode array detector (DAD).

**Table 5:** Linear Regression Data and Limits of Detection (LOD) and Quantification (LOQ) for the Amino Acid Calibration Curve

Standard	Regression equation (Y = aX + b)	Correlation coefficient (*r* ^2^)	Linear range (pmol·μL^–1^)	LOD (pmol·μL^–1^)	LOQ (pmol·μL^–1^)
Asp	Y = 6629X-1903	0.9941	4.0 – 10.0	0.37	1.11
Ser	Y = 11195X-4479	0.9953	4.0 – 10.0	0.21	0.64
Glu	Y = 6549X-3244	0.9996	4.0 – 10.0	0.37	1.13
Gly	Y = 8000X-2997	0.9985	4.0 – 10.0	0.32	0.97
His	Y = 8281X-1878	0.9984	4.0 – 10.0	0.31	0.94
Arg	Y = 8929X+3119	0.9923	4.0 – 10.0	0.14	0.42
Thr	Y = 7465X-405	0.9978	4.0 – 10.0	0.15	0.45
Ala	Y = 1338X+126	0.9975	4.0 – 10.0	0.22	0.67
Pro	Y = 1471X-471	0.9984	4.0 – 10.0	0.14	0.43
Cys	Y = 7140X+740	0.9983	4.0 – 8.0	0.17	0.53
Tyr	Y = 8430X+1498	0.9971	4.0 – 10.0	0.14	0.44
Val	Y = 7254X+1184	0.9967	4.0 – 8.0	0.20	0.60
Met	Y = 8160X-2921	0.9988	4.0 – 10.0	0.17	0.52
Lys	Y = 11149X+3199	0.9978	4.0 – 10.0	0.13	0.40
Ile	Y = 7797X+4654	0.9992	4.0 – 8.0	0.14	0.42
Leu	Y = 8057X+19	0.9978	4.0 – 10.0	0.12	0.35
Phe	Y = 8656X+1090	0.9979	4.0 – 10.0	0.07	0.22

The variability of amino acids among pollen samples was investigated using principal component analysis (PCA) following the same approach applied to physicochemical parameters and mineral concentrations. Initially, PCA (Figure 7) was performed considering the amino acid content of all pollen samples, and the results are presented as scores (Figure 7a) and loadings (Figure 7b). The loadings indicated that sample BP04 exhibited markedly higher concentrations of most amino acids, corresponding to the highest positive scores on PC1 (Figure 7a). Therefore, this sample was classified as an outlier, lying outside the normal distribution, considering a 95% confidence level. Samples BP05, BP11, and BP12 also showed relatively high amino acid concentrations, although they were lower than those of BP03.

**Figure 7:**
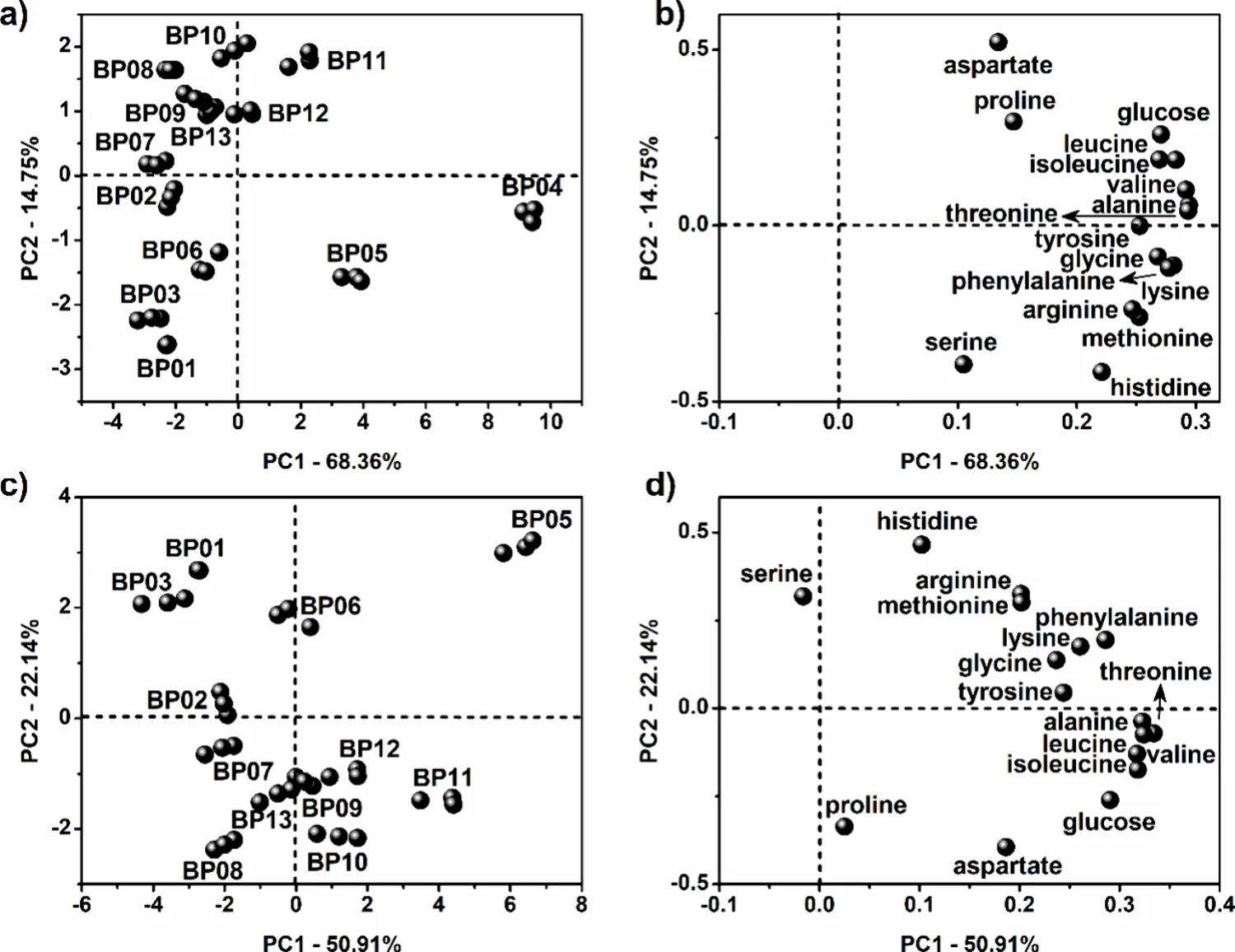
a) PC1 × PC2 scores coordinate system from the evaluation of amino acid variability considering all the pollen samples; b) respective PC1 × PC2 loadings; c) PC1 × PC2 scores from the evaluation of amino acids excluding outlier samples (named as 06.12); d) respective PC1 × PC2 loadings.

To better evaluate the variability among the remaining samples, a second PCA was performed, excluding the outlier sample (BP04). The corresponding scores and loadings are shown in Figures 7c and 7d. After the removal of this sample, the PCA results highlighted elevated concentrations of most amino acids, except serine and proline, in samples with positive PC1 scores, namely, BP05, BP09, BP10, BP11, and BP12.

Quantitative differences among samples were evaluated using one-way ANOVA. Table 4 presents the ANOVA results, in which different letters indicate statistically significant differences among samples. The ANOVA results corroborated and complemented the multivariate analysis, as some variability patterns were more clearly revealed by PCA, whereas others were better evidenced by univariate statistical analysis. As shown in Table 4, the higher concentrations of almost all amino acids in sample BP04 were statistically confirmed by ANOVA, with the exception of aspartate, serine, and proline. In addition, sample BP05 presented elevated levels of several amino acids including histidine, arginine, alanine, tyrosine, valine, methionine, leucine, and phenylalanine. Elevated concentrations of these amino acids were also observed, although to a lesser extent, in samples BP09, BP10, BP11, and BP12, confirming the variability in the amino acid profiles of the analyzed bee pollen samples. Overall, the amino acid profile highlights the nutritional value of the analyzed bee pollen and reinforces its potential as a functional food source.

### 3.5 Sugar Analysis by High-Performance Liquid Chromatography with Refractive Index Detection (HPLC-RID)

The sugar composition of bee pollen was determined using high-performance liquid chromatography with refractive index detection (HPLC-RID). Table 6 presents a quantitative analysis of the two major sugars, glucose and fructose. The combined content of these predominant monosaccharides ranged from 32.52 to 51.59%. The fructose content was equal to or slightly higher than glucose, resulting in a fructose/glucose (F/G) ratio ranging from 1.01 to 1.47. Similar values have been reported for bee pollen samples, with F/G ratios between 1.20 and 1.50.^66^ These results are consistent with previous studies reporting fructose and glucose as the predominant sugars in bee pollen. Wu et al. reported fructose and glucose concentrations of 10.9–12.6% and 6.8–9.6%, respectively, in 80 bee pollen samples analyzed by HPLC-RID.^67^ More recently, HPLC-RID analyses of bee pollen also confirmed the predominance of these two monosaccharides, although their concentrations varied according to botanical and geographical origin.^68,69^ Monosaccharides account for approximately 94% of the total sugar content in bee pollen,^70^ and the high proportion of reducing sugars distinguishes bee pollen from other plant pollen.^58^ The HPLC-RID chromatogram of coconut bee pollen is presented in Figure 8. The calibration curves for glucose and fructose showed excellent linearity over the concentration range of 1.25–20.0 mg mL^–1^, with correlation coefficients (*r* ^2^) of 0.9993 and 0.9987, respectively. The limits of detection (LOD) were 0.006 mg mL^–1^ for both glucose and fructose, while the limits of quantification (LOQ) were 0.017 and 0.019 mg mL^–1^, respectively. The regression equations, linear ranges, LOD, and LOQ values obtained for glucose and fructose are summarized in Table 7.

**Table 6:** Sugar Composition of Coconut (*Cocos nucifera*) Bee Pollen Produced by *Apis mellifera* ^a^

**Samples**	**Glucose (%)**	**Fructose (%)**	F/G	**G + F (%)**
BP01	20.65 ± 1.09	22.06 ± 0.40	1.07	42.70
BP02	15.51 ± 0.69	17.00 ± 0.72	1.10	32.52
BP03	22.45 ± 0.32	22.62 ± 0.21	1.01	45.07
BP04	19.82 ± 0.10	20.00 ± 0.13	1.01	39.83
BP05	18.03 ± 0.55	19.48 ± 0.42	1.08	37.51
BP06	24.90 ± 1.27	26.69 ± 1.03	1.07	51.59
BP07	21.05 ± 0.18	23.93 ± 0.29	1.14	44.99
BP08	19.99 ± 0.82	28.09 ± 0.57	1.41	48.08
BP09	20.89 ± 0.54	25.48 ± 0.51	1.22	46.38
BP10	17.49 ± 0.58	25.75 ± 0.82	1.47	43.24
BP11	19.59 ± 0.71	28.43 ± 0.72	1.45	48.02
BP12	21.30 ± 0.46	29.42 ± 0.68	1.38	50.72
BP13	18.79 ± 0.53	27.42 ± 0.57	1.46	46.21
**Average ± SD**				**45.07 ± 5.32**

a mean ± SD.

**Figure 8:**
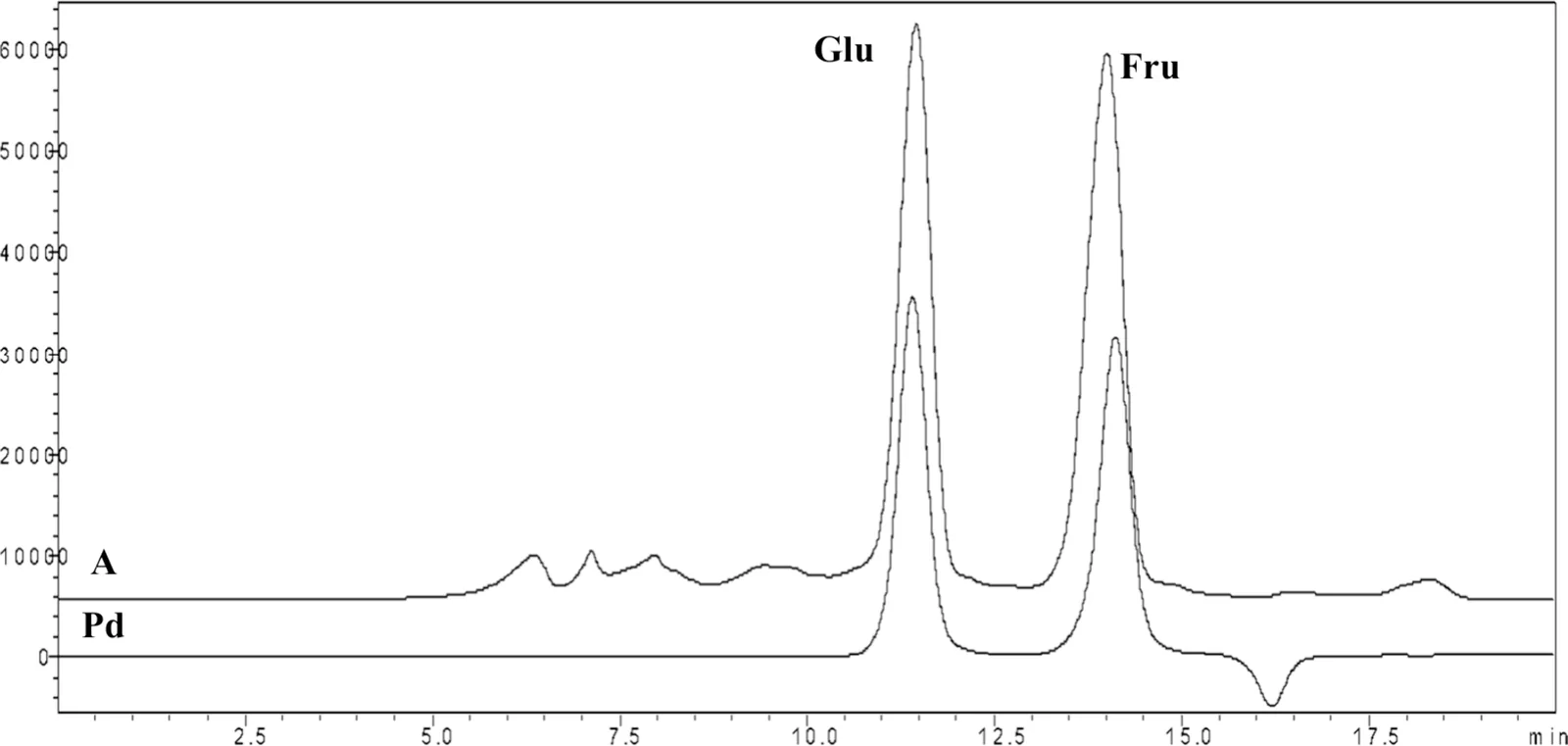
HPLC-RID chromatograms of sugar standards (Pd) and a coconut (*Cocos nucifera*) bee pollen sample produced by *Apis mellifera* (S). The identified monosaccharides were glucose (Glu) and fructose (Fru). Chromatographic conditions: Rezex RCM Monosaccharide Ca^2+^ (8%) column (Phenomenex, 300 mm × 7.8 mm, 8 μm); column temperature 85 °C; flow rate 0.6 mL min^–1^. The mobile phase consisted of water containing 5% methanol and was operated in isocratic mode. HPLC (Shimadzu Prominence LC-20AT, Shimadzu, Kyoto, Japan), LC-6AT pump, DGU-20As degasser, CTO-20AC column oven, autosampler (SIL-20A), and refractive index detector (RID).

**Table 7:** Linear Regression Parameters and Limits of Detection (LOD) and Quantification (LOQ) for the Calibration Curves of Glucose and Fructose in Coconut (*Cocos nucifera*) Bee Pollen Produced by *Apis mellifera*

**Standard**	**Regression equation (Y = aX + b)**	**Correlation coefficient (** ***r*** ^***2***^ **)**	**Linear range (mg·mL** ^**‑1**^ **)**	**LOD (mg·mL** ^**‑1**^ **)**	**LOQ (mg·mL** ^**‑1**^ **)**
Glucose	Y = 178971X+32760	0.9993	1.25 – 20.0	0.006	0.017
Fructose	Y = 192422X-1213	0.9987	1.25 – 20.0	0.006	0.019

### 3.6 Structural Identification of the Major Flavonoid and Characterization of Minor Flavonoids in Cocos nucifera Bee Pollen

The major flavonoid isolated from *Cocos nucifera* bee pollen was identified based on the analysis of ^1^H NMR and APT NMR spectra (Figures 9 and 10, respectively), together with high-resolution mass spectrometry data (Figure 11), as isorhamnetin-3-*O*-neohesperidoside [3-*O*-α-L-rhamnopyranosyl-(1→2)-β-d-glucopyranosyl isorhamnetin] (compound 3, deprotonated molecular ion at *m*/*z* 623.1593 [M-H]^−^).

**Figure 9:**
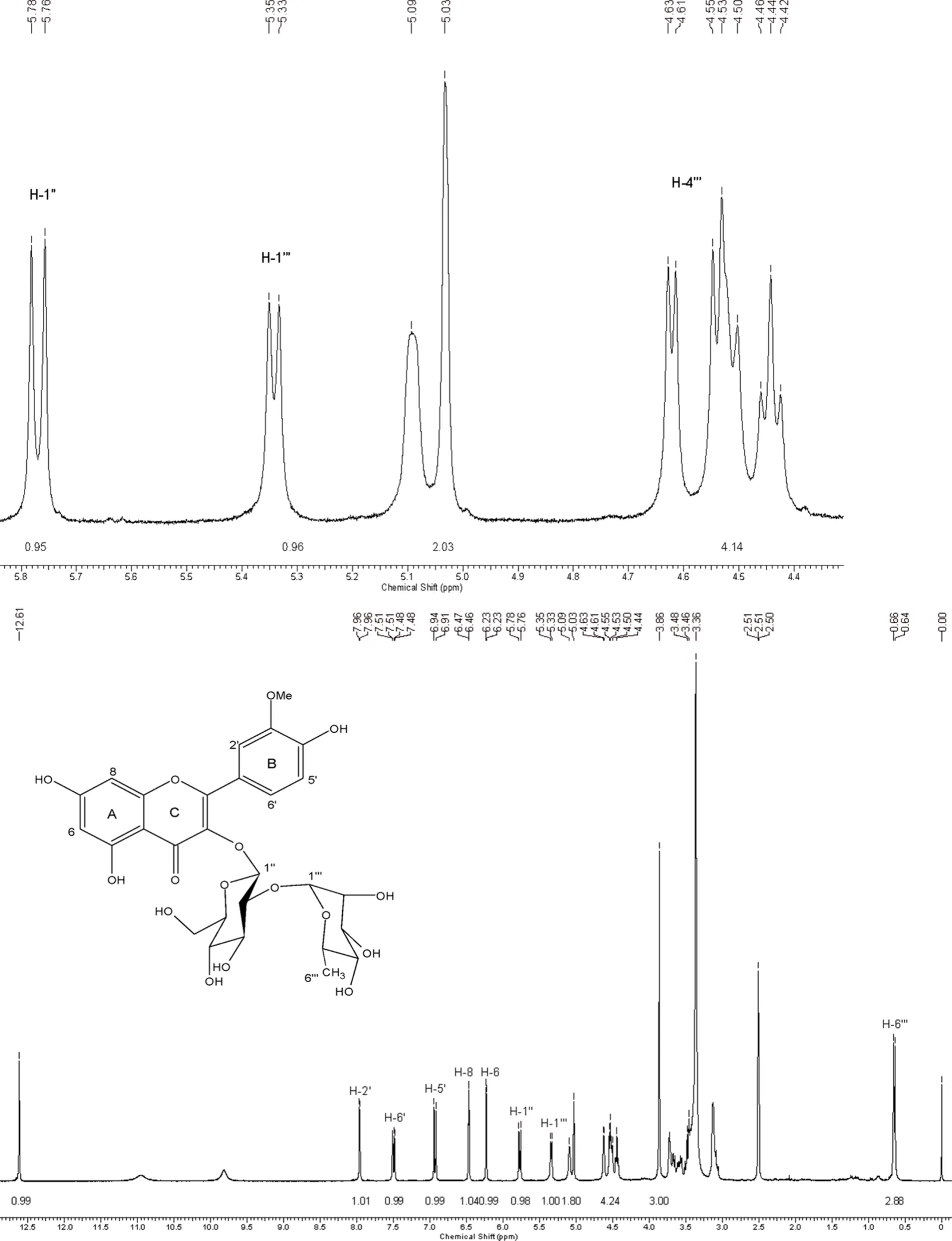
^1^H NMR spectrum (300 MHz) of the flavonoid isorhamnetin-3-*O*-neohesperidoside in DMSO-*d*_*6*_.

**Figure 10:**
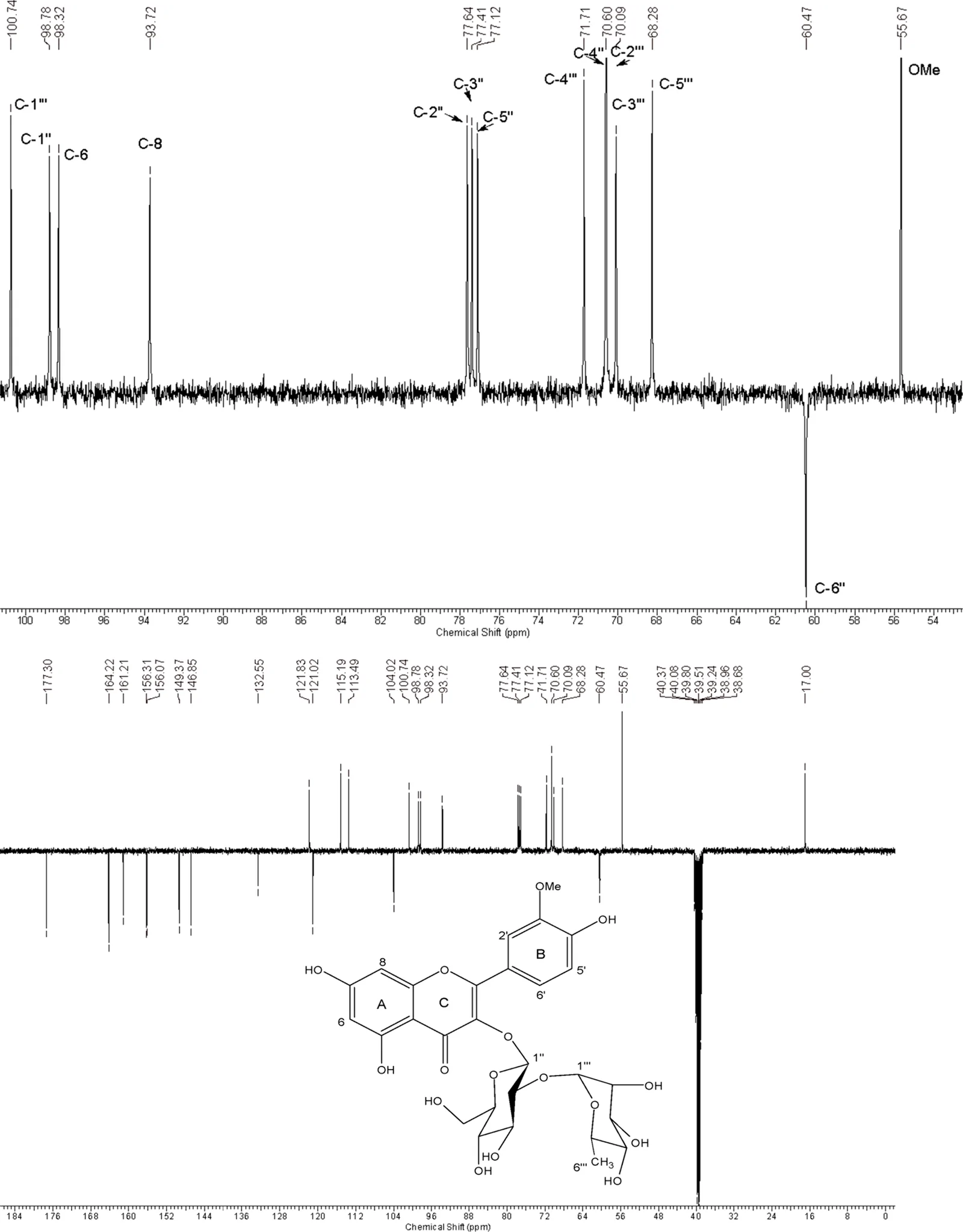
APT spectrum (75 MHz) of the flavonoid isorhamnetin-3-*O*-neohesperidoside in DMSO-*d*6.

**Figure 11:**
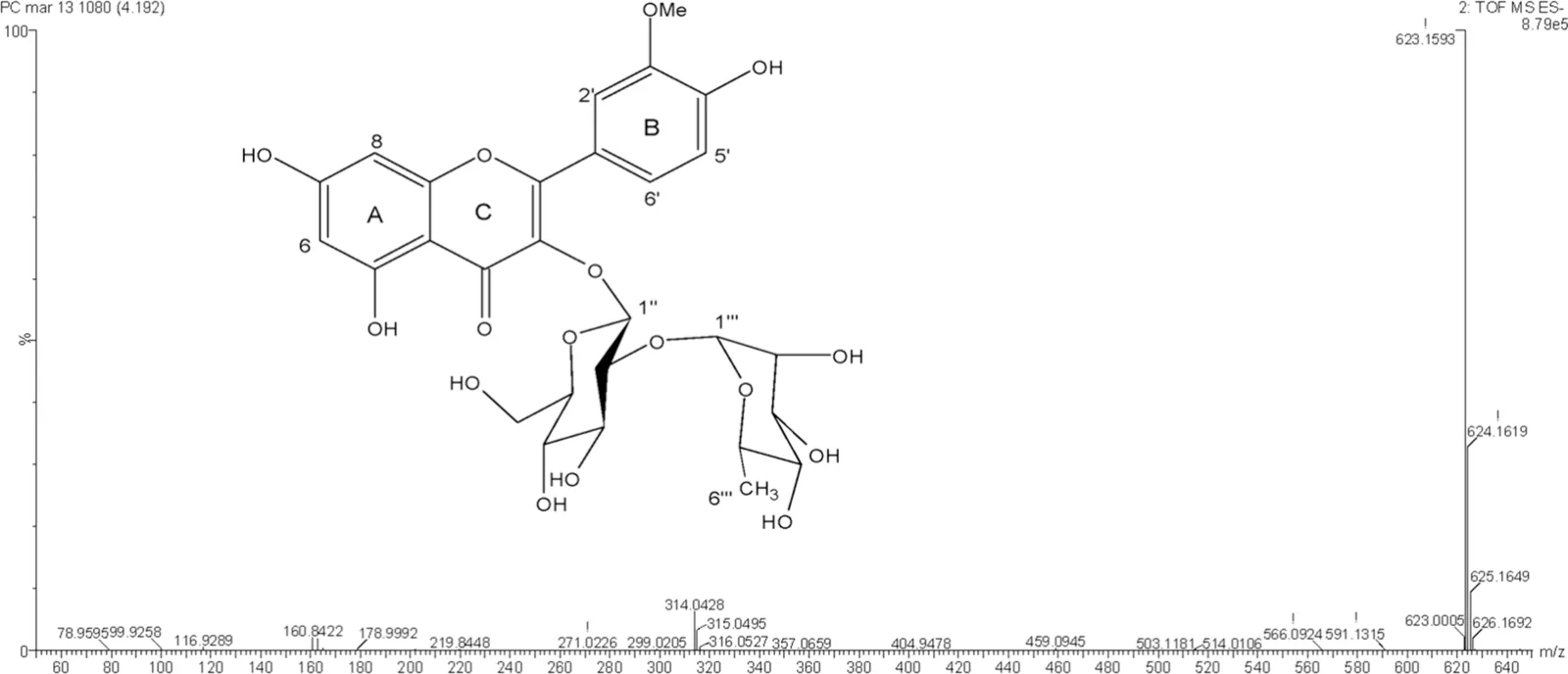
Tandem MS spectra of flavonoid isorhamnetin-3-*O*-neohesperidoside in the negative mode, sensitivity mode, capillary voltage, 3.0 kV; sample cone voltage 40 V, source temperature, 120 °C; desolvation temperature, 450 °C; cone gas flow rate, 50 L/h; and desolvation gas (N2) flow rate, 800 L/h. High energy, ramp collision energy from 20 to 30 V. The analyses were performed using a XEVO-G2XSQTOF mass spectrometer (Waters, Manchester, UK) connected to an ACQUITY UPLC system (Waters, Milford, MA, USA).

The methanolic fractions obtained by solid-phase extraction (SPE), from which this compound was isolated, were further analyzed by HPLC-DAD (Figure 12) and UPLC-DAD-qTOF-MS^E^ in negative ionization mode. This analysis allowed the detection of three additional minor glycosylated flavonoids in the 13 analyzed samples (Figure 13): quercetin rutinoside (1), isorhamnetin-neohesperidoside-rhamnose (2), and acetylated isorhamnetin-3-*O*-neohesperidoside (4). The structures were proposed based on the interpretation of the product ion spectra (MS^E^) and UV spectra, together with a comparison with literature data.^9,38^

**Figure 12:**
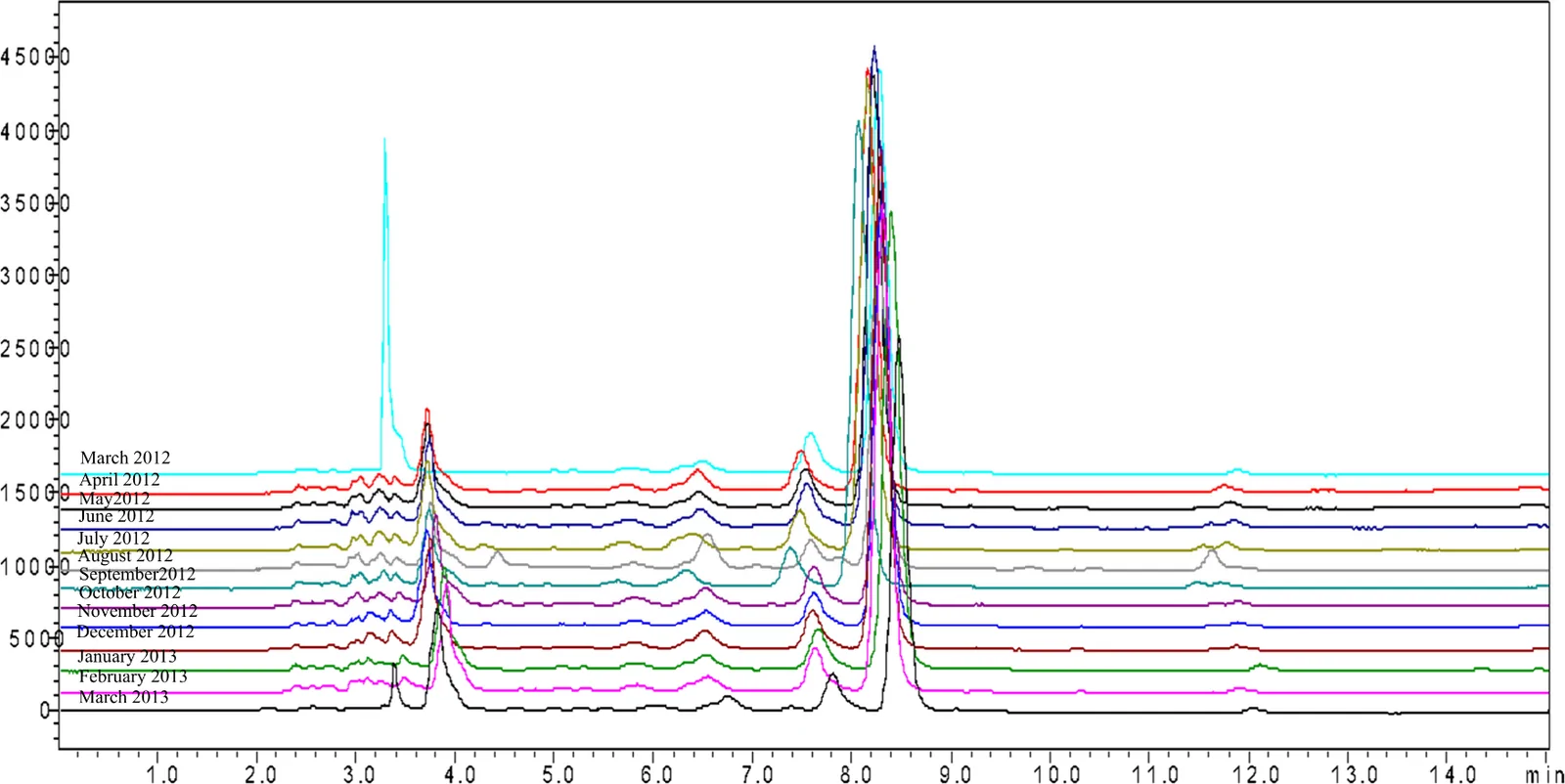
HPLC-DAD chromatograms of the methanolic SPE fraction obtained from coconut (*Cocos nucifera*) bee pollen produced by *Apis mellifera*. Chromatographic conditions: Synergy Hydro-RP column (Phenomenex, 250 mm × 4.6 mm, 5 μm); column temperature 40 °C; flow rate 1.0 mL min^–1^. The mobile phase consisted of water (eluent A) and methanol (eluent B), both acidified with 1% formic acid. The gradient program was as follows: 0–10 min, 40–55% B; 10–15 min, 55–100% B; 15–18 min, 100% B; and 18–25 min, 40% B. HPLC (Shimadzu Prominence LC-20AT, Shimadzu, Kyoto, Japan), two LC-6AT pumps, a DGU-20As degasser, a CTO-20AC oven, and an SPDM-20A diode array detector (DAD).

**Figure 13:**
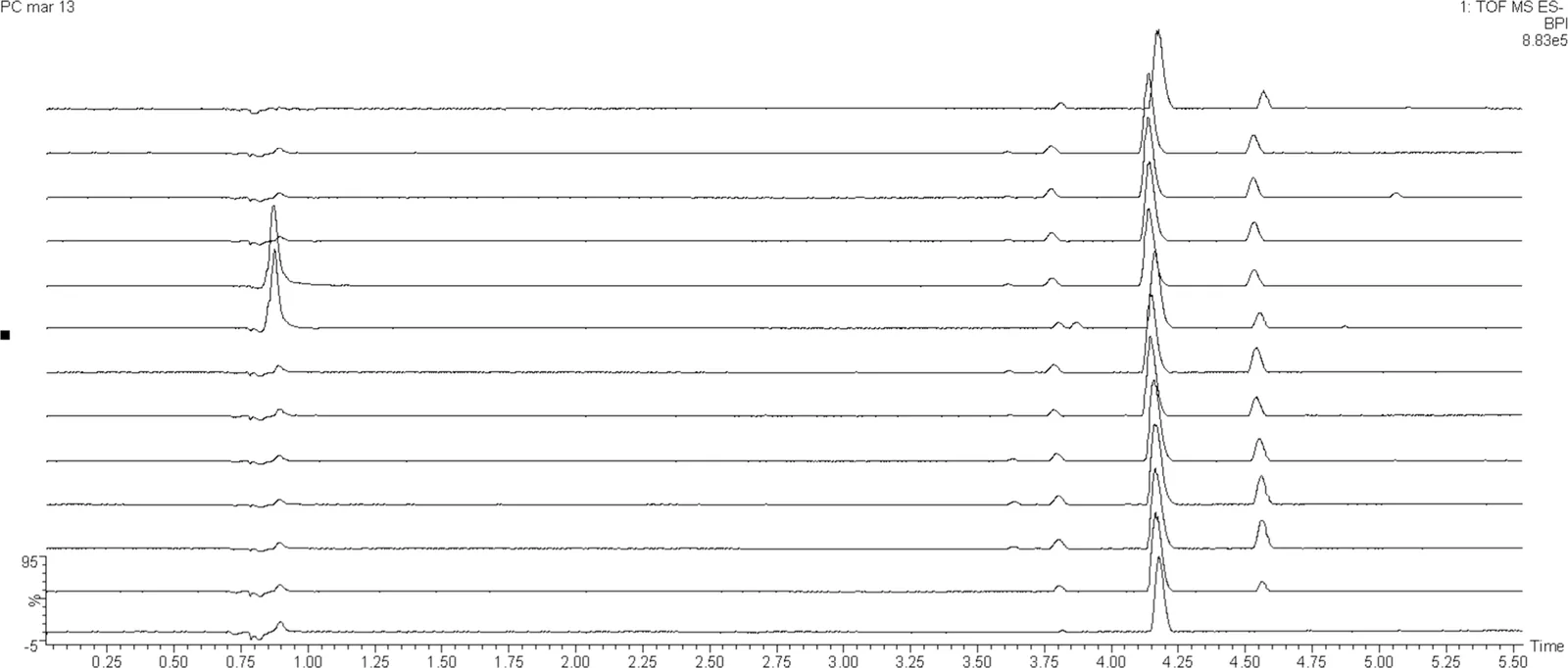
Base peak ion (BPI) chromatograms of the methanolic SPE fraction obtained from coconut (*Cocos nucifera*) bee pollen produced by *Apis mellifera*. Conditions: BEH C-18 Waters column (2.1 mm × 50 mm, 1.7 μm); mobile phase: H_2_O with 0.1% formic acid (solvent A) and CH_3_CN with 0.1% formic acid (solvent B); flow rate, 0.4 mL/min; gradient elution: 0**-**8 min, 10**-**80% B; 8**-**9 min, 100% B. Positive mode, low energy, sensitivity mode, capillary voltage, 2.0 kV; sample cone, source temperature, 120 °C; desolvation temperature, 450 °C; cone gas flow rate, 50 L/h; and desolvation gas (N2) flow rate, 800 L/h. The analyses were performed using a XEVO-G2XSQTOF mass spectrometer (Waters, Manchester, UK) connected to an ACQUITY UPLC system (Waters, Milford, MA, USA).

Compound 1 exhibited a deprotonated molecular ion at *m*/*z* 609.1452 [M-H]^−^ and a characteristic product ion at *m*/*z* 301 [M-H-rhamnose-glucose]^−^, corresponding to the loss of a rutinoside moiety, which allowed its tentative identification as quercetin rutinoside. Compounds 2 and 4 were identified as isorhamnetin derivatives, both showing a characteristic fragment ion at *m*/*z* 315 corresponding to the aglycone. Compound 2 presented a deprotonated molecular ion at *m*/*z* 769.2210 [M-H]^−^ and generated product ions at *m*/*z* 623 [M-H-rhamnose]^−^, *m*/*z* 477 [M-H-2 × rhamnose]^−^, and *m*/*z* 315 [M-H-2 × rhamnose-glucose]^−^, corresponding to the sequential loss of one and two rhamnose and glucose units, respectively. Based on these fragmentation patterns, compound 2 was tentatively identified as isorhamnetin-neohesperidoside-rhamnose. Compound 4 showed a deprotonated molecular ion at *m*/*z* 665.1724 [M-H]^−^. The fragmentation pattern indicated the presence of two sugar units (rhamnose and glucose) together with an acetyl group, producing fragment ions consistent with the sequential loss of rhamnose-glucose and acetyl moieties, yielding the diagnostic ion at *m*/*z* 315 corresponding to the isorhamnetin aglycone. Based on these data, compound 4 was identified as acetylated isorhamnetin-3-*O*-neohesperidoside.

Flavonoids 2, 3, and 4 have been previously reported in bee pollen samples of *Mimosa pudica* collected in the municipality of Nilo Peçanha, Bahia, Brazil.^9^ Compound 3 (isorhamnetin-3-*O*-neohesperidoside) was also identified in monofloral bee pollen from *Attalea funifera* Mart. (Arecaceae), known as piaçava, was collected from the same region.^38^ Additionally, other isorhamnetin glycosides, such as isorhamnetin-3-*O*-(2″-*O*-rhamnosyl)­glucoside (isorhamnetin-3-*O*-neohesperidoside), isorhamnetin-3-*O*-(2″,3″-*O*-dirhamnosyl)­glucoside, and isorhamnetin-di-3,7-*O*-glucoside, have been described in *Apis mellifera* bee pollen samples collected in the municipality of Canavieiras, Bahia State, where *Cocos nucifera* was identified as the predominant pollen taxon (99.0%).^57^ The same compound was isolated by Freire et al.^36^ from bee pollen samples of different botanical origins collected in the same region.

### 3.7 Quantification of the Major Flavonoid Isorhamnetin-3-O-neohesperidoside in Bee Pollen Samples and in Pollen Grains Collected from Cocos nucifera Inflorescences

The major flavonoid identified in *Cocos nucifera* bee pollen, isorhamnetin-3-*O*-neohesperidoside, was quantified in all bee pollen samples and in pollen grains directly collected from *Cocos nucifera* inflorescences. Quantification was performed by HPLC-DAD using the isolated compound as an external standard.

The compound was detected in all analyzed bee pollen samples, with concentrations ranging from 3.20 ± 0.06 mg g^–1^ of pollen (sample BP08) to 4.90 ± 0.20 mg g^–1^ of pollen (sample BP03) with an average concentration of 4.11 ± 0.48 mg g^–1^ of pollen (Table 8). The linear regression parameters of the calibration curves, together with the limits of detection (LOD) and quantification (LOQ), are presented in Table 9. Although small variations in concentration were observed among the samples collected throughout the year, isorhamnetin-3-*O*-neohesperidoside remained the predominant flavonoid in all samples, demonstrating a remarkably consistent presence in coconut bee pollen. This consistency strongly supports the use of this compound as a potential marker for *C. nucifera*-derived bee pollen. To further confirm the botanical origin of this flavonoid, extracts obtained from pollen grains directly collected from *Cocos nucifera* inflorescences were analyzed (Table 8). The concentration of isorhamnetin-3-*O*-neohesperidoside in floral pollen was 0.39 ± 0.03 mg g^–1^ of pollen. In addition to the major compound (8.65 ± 0.64 of μg g^–1^ of extract), the same minor flavonoids, quercetin rutinoside (1), isorhamnetin-neohesperidoside-rhamnose (2), and acetylated isorhamnetin-3-*O*-neohesperidoside (4), previously detected in bee pollen samples, were also observed in the extracts of floral pollen grains (Figure 14). The detection of an identical flavonoid profile in both bee and floral pollen grains provides strong evidence that these compounds originate from *C. nucifera* pollen. This comparative approach reinforces the reliability of flavonoids as potential markers associated with coconut bee pollen.

**Figure 14:**
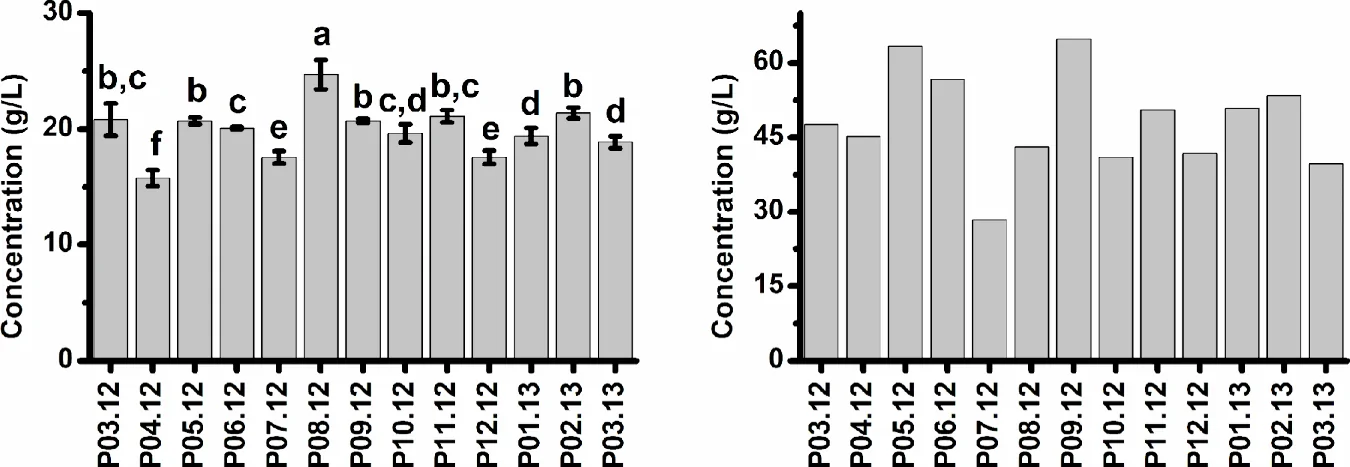
Concentrations of flavonoids in pollen (a) and percentage of pollen from *Cocos nucifera* (b).

**Table 8:** Quantification of Isorhamnetin-3-*O*-neohesperidoside in Samples of Bee and Flower Pollen of *Cocos nucifera* (mg·g^–1^± SD)

**Bee pollen samples**	**Concentration (mg of flav·g** ^**–1**^ **of ext)**	**Concentration (mg of flav·g** ^**–1**^ **of pollen)**
BP01	8.85 ± 0.31	3.28 ± 0.11
BP02	11.70 ± 0.18	4.24 ± 0.06
BP03	11.11 ± 0.44	4.90 ± 0.20
BP04	11.44 ± 0.38	4.26 ± 0.14
BP05	9.94 ± 0.44	4.27 ± 0.19
BP06	11.08 ± 0.13	4.37 ± 0.05
BP07	11.65 ± 0.19	3.80 ± 0.06
BP08	8.45 ± 0.15	3.20 ± 0.06
BP09	11.28 ± 0.21	3.75 ± 0.07
BP10	11.37 ± 0.14	4.44 ± 0.05
BP11	10.47 ± 0.20	4.38 ± 0.08
BP12	10.39 ± 0.18	4.46 ± 0.08
BP13	10.31 ± 0.54	4.09 ± 0.21
Average ± SD	10.62 ± 1.03	4.11 ± 0.48

***Cocos nucifera*** **flower pollen**	**Concentration (μg of flav·g** ^**–1**^ **of ext)**	**Concentration (mg of flav·g** ^**–1**^ **of pollen)**
**Floral pollen**	8.65 ± 0.64	0.39 ± 0.03

**Table 9:** Linear Regression Parameters and Limits of Detection (LOD) and Quantification (LOQ) for the Calibration Curve of the Major Flavonoid, Isorhamnetin-3-*O*-neohesperidoside

**Standard**	**Regression equation (Y = aX + b)**	**Correlation coefficient (** ***r*** ^***2***^ **)**	**Linear range (mg. mL** ^**‑1**^ **)**	**LOD (mg. mL** ^**‑1**^ **)**	**LOQ (mg. mL** ^**‑1**^ **)**
Flavonoid	Y = 581.5X+495.3	0.9992	5.0 – 80.0	0.33	1.00

A one-way ANOVA was performed to evaluate the variability in flavonoid concentrations among the bee pollen samples (Figure 15). Significant differences between samples are indicated by different letters above the bars. The highest flavonoid concentration was observed in sample BP06, whereas the lowest concentration was detected in sample BP02. Palynological analysis (Figure 15b) revealed that the highest percentage of *C. nucifera* pollen occurred in sample BP07, followed by PB03, whereas the lowest percentage was found in sample BP06. These results indicate that flavonoid concentration does not strictly correlate with the proportion of coconut pollen in the samples, suggesting that the compound is consistently present, even when the botanical composition varies slightly.

**Figure 15:**
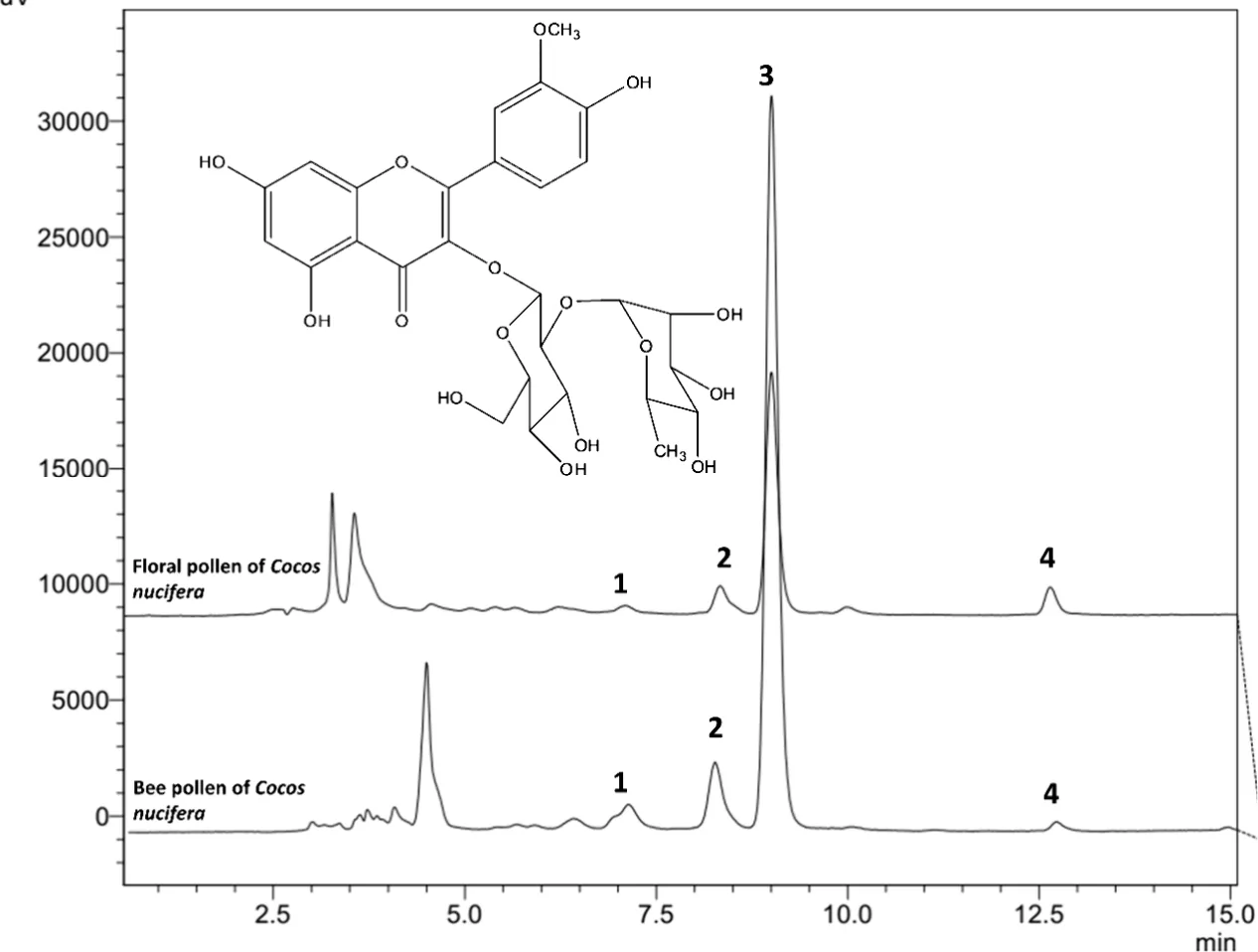
HPLC-DAD chromatograms of coconut (*Cocos nucifera*) bee pollen and floral pollen. The identified flavonoids were quercetin rutinoside (1), isorhamnetin-neohesperidoside-rhamnose (2), isorhamnetin-3-*O*-neohesperidoside (3), and acetylated isorhamnetin-3-*O*-neohesperidoside (4).

## 4 Conclusion

Palynological analysis confirmed the predominance of coconut pollen grains during the study period. The flavonoid isorhamnetin-3-*O*-neohesperidoside was identified as the major phenolic constituent and was detected in both the bee and floral pollens of C. nucifera, supporting its use as a characteristic chemical marker of coconut bee pollen. The concentration of this flavonoid was consistent in samples in which coconut pollen predominated. The physicochemical parameters complied with the Brazilian quality standards for bee pollen. The sugar, free amino acid, and mineral compositions revealed glucose and fructose as the main sugars and potassium, calcium, and magnesium as the most abundant minerals. The combination of multivariate (PCA) and univariate (ANOVA) statistical approaches provided a comprehensive evaluation of the physicochemical and mineral variability of the bee pollen samples, revealing that differences in composition may be associated with variations in environmental conditions and seasonal factors influencing pollen collection. These findings reinforce the importance of combining chemical fingerprinting and palynological analyses as reliable tools for the authentication, compositional characterization, and establishment of quality control parameters, traceability, and authenticity criteria for coconut bee pollen, adding value to this product in the food industry.

## References

[1] Rzetecka N., Matuszewska E., Plewa S., Matysiak J., Klupczynska-Gabryszak A. (2024). Bee products
as valuable nutritional ingredients: determination of broad free amino
acid profiles in bee pollen, royal jelly, and propolis. J. Food Compos. Anal..

[2] Liolios V., Tananaki C., Papaioannou A., Kanelis D., Rodopoulou M. A., Argena N. (2019). Mineral content in
monofloral bee pollen: investigation
of the effect of the botanical and geographical origin. J. Food Meas. Charact..

[3] Anjum S. I., Ullah A., Gohar F., Raza G., Khan M. I., Hameed M., Ali A., Chen C.-C., Tlak
Gajger I. (2024). Bee pollen as a food and feed supplement and a therapeutic remedy:
recent trends in nanotechnology. Front. Nutr..

[4] da
Silva G. R., da Natividade T. B., Camara C. A., da Silva E. M. S., dos Santos F. d. A. R., Silva T. M. S. (2014). Identification
of sugars, amino acids and minerals from the pollen of Jandaíra
stingless bees (*Melipona subnitida*). Food Nutr. Sci..

[5] Lu P., Takiguchi S., Honda Y., Lu Y., Mitsui T., Kato S., Kodera R., Furihata K., Zhang M., Okamoto K., Itoh H., Suzuki M., Kono H., Nagata K. (2022). NMR and HPLC
profiling of bee pollen products from
different countries. Food Chem. Mol. Sci..

[6] Thakur M., Nanda V. (2020). Composition and functionality
of bee pollen: a review. Trends Food Sci. Technol..

[7] Rodríguez-Pólit C., Gonzalez-Pastor R., Heredia-Moya J., Carrera-Pacheco S. E., Castillo-Solis F., Vallejo-Imbaquingo R., Barba-Ostria C., Guamán L. P. (2023). Chemical properties and biological activity of bee
pollen. Molecules.

[8] Zhou W., He Y., Lv J. M., Wang R., He H., Wu M., Zhang R., He J. (2025). Preparation technologies, structural
characteristics and biological activities of polysaccharides from
bee pollen: a review. Int. J. Biol. Macromol..

[9] Pereira
Gomes A. N., Camara C. A., Sousa A. d. S., dos
Santos F. d. A. R., Campos M. G., Silva T. M. S. (2024). Chemical composition
and free radical-scavenging activities of monofloral bee pollen from *Mimosa pudica* L. J. Apic. Res..

[10] Morgano M. A., Martins M. C. T., Rabonato L. C., Milani R. F., Yotsuyanagi K., Rodriguez-Amaya D. B. (2012). A Comprehensive Investigation of the Mineral Composition
of Brazilian Bee Pollen: Geographic and seasonal variations and contribution
to human diet. J. Braz. Chem. Soc..

[11] de
Arruda V. A. S., Pereira A. A. S., de Freitas A. S., Barth O. M., de Almeida-Muradian L.
B. (2013). Dried bee pollen: b
complex vitamins, physicochemical and botanical composition. J. Food Compos. Anal..

[12] Baky M. H., Abouelela M. B., Wang K., Farag M. A. (2023). Bee pollen and bread
as a super-food: a comparative review of their metabolome composition
and quality assessment in the context of best recovery conditions. Molecules.

[13] Bogdanov, S. Pollen: nutrition, functional properties, health. In Pollen Book; Chapter 2; Bee Product Science, 2016. https://freethebees.ch/wp-content/uploads/2020/06/PollenNutritional.pdf (accessed January 13, 2026).

[14] Soares
de Arruda V. A., Vieria dos Santos A., Figueiredo Sampaio D., da Silva Araujo E., de Castro Peixoto A.
L., Estevinho L. M., de Almeida-Muradian L. B. (2021). Brazilian bee pollen: phenolic content,
antioxidant properties and antimicrobial activity. J. Apic. Res..

[15] El-Seedi H. R., El-Wahed A. A. A., Salama S., Agamy N., Altaleb H. A., Du M., Saeed A., Di Minno A., Wang D., Daglia M., Guo Z., Zhang H., Khalifa S. A. (2024). Natural remedies and health; a review
of bee pollen and bee bread impact on combating diabetes and obesity. Curr. Nutr. Rep..

[16] El
Ghouizi A., Bakour M., Laaroussi H., Ousaaid D., El Menyiy N., Hano C., Lyoussi B. (2023). Bee pollen
as functional food: insights into its composition and therapeutic
properties. Antioxidants.

[17] Otręba M., Marek Ł., Stojko J., Rzepecka-Stojko A. (2026). Bee products
as alternatives in the treatment of viral infections. J. Sci. Food Agric..

[18] Nainu F., Masyita A., Bahar M. A., Raihan M., Prova S. R., Mitra S., Emran T. B., Simal-Gandara J. (2021). Pharmaceutical
prospects of bee products: special focus on anticancer, antibacterial,
antiviral, and antiparasitic properties. Antibiotics.

[19] Li Q., Sun M., Wan Z., Liang J., Betti M., Hrynets Y., Xue X., Wu L., Wang K. (2019). Bee pollen extracts modulate serum
metabolism in lipopolysaccharide-induced acute lung injury mice with
anti-inflammatory effects. J. Agric. Food Chem..

[20] Kassianenko V. I., Komisarenko I. A., Dubtsova E. A. (2011). Correction of atherogenic dyslipidemia
with honey, pollen and bee bread in patients with different body mass. Ter. Arkh..

[21] Giampieri F., Quiles J. L., Cianciosi D., Forbes-Hernández T. Y., Orantes-Bermejo F. J., Alvarez-Suarez J. M., Battino M. (2022). Bee products: an emblematic
example of underutilized sources of bioactive compounds. J. Agric. Food Chem..

[22] Ishikawa Y., Tokura T., Nakano N., Hara M., Niyonsaba F., Ushio H., Yamamoto Y., Tadokoro T., Okumura K., Ogawa H. (2008). Inhibitory effect of
honeybee-collected pollen on mast cell degranulation. J. Med. Food.

[23] Ishikawa Y., Tokura T., Ushio H., Niyonsaba F., Yamamoto Y., Tadokoro T., Ogawa H., Okumura K. (2009). Lipid-soluble
components of honeybee-collected pollen exert antiallergic effect
by inhibiting IgE-mediated mast cell activation *in vivo*. Phytother. Res..

[24] Khalifa S. A. M., Elashal M. H., Yosri N., Du M., Musharraf S. G., Nahar L., Sarker S. D., Guo Z., Cao W., Zou X., Abd El-Wahed A. A., Xiao J., Omar H. A., Hegazy M.-E. F., El-Seedi H. R. (2021). Bee pollen: current status and therapeutic
potential. Nutrients.

[25] Kacemi R., Campos M. G. (2025). Bee pollen phytochemicals
and nutrients as unequaled
pool of epigenetic regulators: implications for age-related diseases. Foods.

[26] da
Silva de Freitas A., Sattler J. A. G., de Souza B. R., Almeida-Muradian L. B., Sattler A., Barth O. M. (2015). A melissopalynological analysis of
Apis mellifera L. loads of dried bee pollen in the southern Brazilian
macro-region. Grana.

[27] Ansaloni L. S. (2025). An overview of the nutritional
requirements of honey bees (*Apis mellifera* Linnaeus,
1758). Insects.

[28] Marchini L. C., Reis V. D. A., Moreti A. C. C. C. (2006). Composição físico-química
de amostras de pólen. Ciênc. Rural.

[29] Barbieri D., Gabriele M., Summa M., Colosimo R., Leonardi D., Domenici V., Pucci L. (2020). Antioxidant, nutraceutical properties,
and fluorescence spectral profiles of bee pollen samples from different
botanical origins. Antioxidants.

[30] Li Q. Q., Wang K., Marcucci M. C., Sawaya A. C. H. F., Hu L., Xue X. F., Wu L. M., Hu F. L. (2018). Nutrient-rich bee
pollen: a treasure trove of active natural metabolites. J. Funct. Foods.

[31] Alcalá-Orozco M., Lobo-Farfan I., Tirado D. F., Mantilla-Escalante D. C. (2024). Enhancing
the nutritional and bioactive properties of bee pollen: a review of
processing techniques. Foods.

[32] Végh R., Csóka M., Sörös C., Sipos L. (2021). Food safety hazards
of bee pollen: a review. Trends Food Sci. Technol..

[33] Santisteban R., Cabrera S., Neto J., Silva E., Correia R., Alves R., Santos F., Camara C., Silva T. (2019). Análises
melissopalinológicas, físico-químicas, atividade
antirradicalar e perfil químico por UPLC-DAD-QTOF-MS/MS dos
méis de *Frieseomelitta doederleini* (Abelha
Branca): comparação com os fenólicos presentes
nas flores de *Mimosa tenuiflora* (Jurema Preta). Quim. Nova.

[34] Brazil. Ministry of Agriculture and Livestock . Regulatory Instruction SDA No. 03. MAPA: Brasília, Brazil, 2001. https://www.gov.br/agricultura/pt-br/assuntos/inspecao/produtos-animal/legislacao/IN032001RTprodutosdeabelhas.pdf (accessed January 13, 2026).

[35] Caldas F., Augusto Filho F., Facundo H., Alves R., Santos F., Silva G., Camara C., Silva T. (2018). Composição
química, atividade antirradicalar e antimicrobiana do pólen
apícola de Fabaceae. Quim. Nova.

[36] Freire K. R., Lins A. C., Dórea M. C., Santos F. A., Camara C. A., Silva T. M. (2012). Palynological origin,
phenolic content, and antioxidant
properties of honeybee-collected pollen from Bahia, Brazil. Molecules.

[37] Karam V. M., Silva T. M. S., Santos F. D. A. R. (2022). Pollen
profile of bee pollen from
Semiarid Northeastern Brazil. Acta Bot. Bras..

[38] Gomes A. N. P., Camara C. A., dos Santos
Sousa A., de Assis Ribeiro dos Santos F., de Santana Filho P. C., Dorneles G. P., Romao P. R. T., Silva T. M. S. (2021). Chemical
composition of bee pollen and leishmanicidal activity of Rhusflavone. Rev. Bras. Farmacogn..

[39] Silva T. M. S., Camara C. A., da Silva
Lins A. C., Maria Barbosa-Filho J., da Silva E. M. S., Freitas B. M., de Assis Ribeiro dos Santos F. (2006). Chemical composition
and free radical scavenging activity of pollen loads from stingless
bee *Melipona subnitida* Ducke. J. Food Compos. Anal..

[40] Silva T., Camara C. A., Lins A., Agra M. F., Silva E., Reis I. T., Freitas B. M. (2009). Chemical
composition, botanical evaluation
and screening of radical scavenging activity of collected pollen by
the stingless bees *Melipona rufiventris* (Uruçu-Amarela). An. Acad. Bras. Ciênc..

[41] de
Souza S. A., da Silva T. M. G., da Silva E. M. S., Camara C. A., Silva T. M. S. (2018). Characterisation of phenolic compounds by UPLC-QTOF-MS/MS
of geopropolis from the stingless bee *Melipona subnitida* (Jandaíra). Phytochem. Anal..

[42] Medeiros K.C.P., Figueiredo C.A.V., Figueredo T.B., Freire K.R.L., Santos F.A.R., Alcantara-Neves N.M., Silva T.M.S., Piuvezam M.R. (2008). Anti-allergic effect
of bee pollen phenolic extract and myricetin in ovalbumin-sensitized
mice. J. Ethnopharmacol..

[43] Alvarado J. L., Delgado-Rueda M. D. (1985). Flora Apícola
en Uxpanapa, Veracruz, México. Biotica.

[44] de
Novais J. S., Cristina Lima e Lima L., de Assis
Ribeiro dos Santos F. (2009). Botanical affinity of pollen harvested by *Apis
mellifera*. Grana.

[45] Erdtman G. (1960). The acetolysis
method: a revised description. Sven. Bot. Tidskr..

[46] Jones, G. D. ; Bryant, V. M. Melissopalynology. In Palynology: Principles and Applications; Jansonius, J. ; McGregor, D. C. , Eds.; American Association of Stratigraphic Palynologists Foundation: Dallas, TX, 1996; Vol. 3, pp 933–938.

[47] Santisteban R. M., Fontoura D. M. N. d.
S., Carvalho
de Jesus M., Santos F. d. A. R. d., Camara C. A., de Sousa L. H. N., Alves Filho E. d. G., Sarmento da Silva E. M., Cunha A. N., Silva T. M. S. (2025). Melissopalinological
analysis, physicochemical properties, and chemical composition of *Apis mellifera* and *Melipona mandacaia* honey
from the Brazilian Semi-Arid Region. ACS Food
Sci. Technol..

[48] Instituto Adolfo Lutz . Métodos Físico-Químicos para Análise de Alimentos, 4th ed.; Instituto Adolfo Lutz: São Paulo, Brazil, 2008. https://www.ial.sp.gov.br/resources/editorinplace/ial/2016_3_19/analisedealimentosial_2008.pdf?attach=true (accessed January 13, 2026).

[49] da
Silva G. R., da Natividade T. B., Camara C. A., da Silva E. M. S., dos Santos F. d. A. R., Silva T. M. S. (2014). Identification
of sugars, amino acids and minerals from the pollen of Jandaíra
stingless bees (*Melipona subnitida*). Food Nutr. Sci..

[50] Bro R., Smilde A. K. (2014). Principal Component
Analysis. Anal. Methods.

[51] de
Sousa-Fontoura D. M. N., de Amorim Silva P. H.
X., de Assis
Ribeiro dos Santos F., Camara C. A., Nogueira de Sousa L. H., de Godoy Alves Filho E., Araujo R. M., Lima Y. S., Sarmento
da Silva E. M., Sarmento Silva T. M. (2025). Polyphenolic profile, botanical origin,
and radical scavenging activity of geopropolis produced by the stingless
bee *Melipona subnitida* Ducke in Northeastern Brazil. Food Res. Int..

[52] Lorenzi, H. Árvores Brasileiras: Manual de Identificação e Cultivo de Plantas Arbóreas do Brasil, 2nd ed.; Instituto Plantarum: Nova Odessa, Brazil, 2002; Vol. 2.

[53] Bogdanov, S. Pollen Book, Chapter 1; Bee Product Science, 2014. https://www.bee-hexagon.net (accessed January 13, 2026).

[54] Estevinho L. M., Rodrigues S., Pereira A. P., Feás X. (2012). Portuguese
bee pollen: palynological study, nutritional and microbiological evaluation. Int. J. Food Sci. Technol..

[55] Morais M., Moreira L., Feás X., Estevinho L. M. (2011). Honeybee-collected
pollen from five portuguese natural parks: palynological origin, phenolic
content, antioxidant properties, and antimicrobial activity. Food Chem. Toxicol..

[56] Komosinska-Vassev K., Olczyk P., Kaźmierczak J., Mencner L., Olczyk K. (2015). Bee pollen:
chemical composition and therapeutic application. Evid. Based Complement. Alternat. Med..

[57] Negri G., Barreto L. M. R. C., Sper F. L., Carvalho C., Campos M. G. R. (2018). Phytochemical
analysis and botanical origin of *Apis mellifera* bee
pollen from the municipality of Canavieiras, Bahia State, Brazil. Braz. J. Food Technol..

[58] Li Q. Q., Liang X., Xue X., Wang K., Wu L. (2020). Lipidomics
provides novel insights into understanding the bee pollen lipids transepithelial
transport and metabolism in human intestinal cells. J. Agric. Food Chem..

[59] Carpes S. T., Prado A., Moreno I. A. M., Mourão G. B., Alencar S. M., Masson M. L. (2008). Screening of the antioxidant potential
of bee pollen produced in the Southern Region of Brazil. Quim. Nova.

[60] Martins M. C. T., Morgano M. A., Vicente E., Baggio S., Rodriguez-Amaya D. B. (2011). Physicochemical
composition of bee pollen from eleven Brazilian states. J. Apic. Sci..

[61] Human H., Nicolson S. W. (2006). Nutritional content of fresh, bee-collected and stored
pollen of *Aloe greatheadii* var. *davyana* (Asphodelaceae). Phytochemistry.

[62] de
Arruda V. A. S., Vieria dos Santos A., Figueiredo Sampaio D., da Silva Araujo E., de Castro Peixoto A.
L., Estevinho M. L. F., Bicudo de Almeida-Muradian L. (2017). Microbiological Quality and Physicochemical
Characterization of Brazilian Bee Pollen. J.
Apic. Res..

[63] Dominguez-Valhondo D., Bohoyo Gil D., Hernandez M. T., Gonzalez-Gomez D. (2011). Influence
of the commercial processing and floral origin on bioactive and nutritional
properties of honeybee-collected pollen. Int.
J. Food Sci. Technol..

[64] Dutra A. T. B., Silva E. N., Rodrigues C. R. F., Vieira S. A., Aragão R. M., Silveira J. A. (2011). High temperatures
affect ion distribution in cowpea
plants pre-treated with NaCl. Rev. Bras. Eng.
Agríc. Ambient..

[65] Machado
De-Melo A. A., Almeida-Muradian L. B.
d., Sancho M. T., Pascual-Mate A. (2018). Composition and properties of *Apis mellifera* honey: a review. J. Apic. Res..

[66] Bobis O., Moise A. R., Ballesteros I., Reyes E. S., Durán S. S., Sánchez-Sánchez J., Cruz-Quintana S., Giampieri F., Battino M., Alvarez-Suarez J. M. (2020). Eucalyptus
honey: quality parameters, chemical composition and health-promoting
properties. Food Chem..

[67] Wu L.-M., Xue X.-F., Zhou X., Chen F., Zhao J. (2008). Determination
of sugars in bee pollen by HPLC. Food Sci..

[68] Oroian M., Dranca F., Ursachi F. (2022). Characterization
of Romanian bee
pollen-An important nutritional source. Foods.

[69] Yıldırım N., Gerçek Y. C. (2025). Preliminary
characterization of monofloral Helianthus
annuus L. bee pollen using advanced analytical techniques: A comparative
study with polyfloral pollen. Food Sci. Nutr..

[70] Kieliszek M., Piwowarek K., Kot A. M., Błażejak S., Chlebowska-Śmigiel A., Wolska I. (2018). Pollen and bee bread
as new health-oriented products: a review. Trends
Food Sci. Technol..

